# The bHLH Subgroup IIId Factors Negatively Regulate Jasmonate-Mediated Plant Defense and Development

**DOI:** 10.1371/journal.pgen.1003653

**Published:** 2013-07-25

**Authors:** Susheng Song, Tiancong Qi, Meng Fan, Xing Zhang, Hua Gao, Huang Huang, Dewei Wu, Hongwei Guo, Daoxin Xie

**Affiliations:** 1 Tsinghua-Peking Center for Life Sciences, MOE Key Laboratory of Bioinformatics, School of Life Sciences, Tsinghua University, Beijing, China; 2 State Key Laboratory of Protein and Plant Gene Research, College of Life Sciences, Peking-Tsinghua Center for Life Sciences, Peking University, Beijing, China; National University of Singapore and Temasek Life Sciences Laboratory, Singapore

## Abstract

Plants have evolved sophisticated systems for adaptation to their natural habitat. In response to developmental and environmental cues, plants produce and perceive jasmonate (JA) signals, which induce degradation of JASMONATE-ZIM-Domain (JAZ) proteins and derepress the JAZ-repressed transcription factors to regulate diverse aspects of defense responses and developmental processes. Here, we identified the bHLH subgroup IIId transcription factors (bHLH3, bHLH13, bHLH14 and bHLH17) as novel targets of JAZs. These bHLH subgroup IIId transcription factors act as transcription repressors and function redundantly to negatively regulate JA responses. The quadruple mutant *bhlh3 bhlh13 bhlh14 bhlh17* showed severe sensitivity to JA-inhibited root growth and JA-induced anthocyanin accumulation, and exhibited obvious increase in JA-regulated plant defense against pathogen infection and insect attack. Transgenic plants overexpressing *bHLH13* or *bHLH17* displayed reduced JA responses. Furthermore, these bHLH factors functioned as transcription repressors to antagonize the transcription activators, such as MYC2 and the WD-repeat/bHLH/MYB complex, through binding to their target sequences. Coordinated regulation of JA responses by transcription activators and repressors would benefit plants by allowing fine regulation of defense and development, and survival in their frequently changing environment.

## Author Summary

Plants live in fixed places and have to evolve sophisticated systems for adaptation to their frequently changing environment. Plant hormones are essential for the regulation of these sophisticated systems which coordinately control plant growth, development, reproduction and defense. Jasmonates (JAs), a new class of cyclic fatty acid-derived plant hormone, regulate diverse aspects of plant defense and developmental processes. In response to external environmental signals and internal developmental cues, plants rapidly produce and efficiently perceive JA signals, which regulate a dynamic regulatory network to activate various downstream transcription factors essential for appropriate plant defense and development. Here, we identified the bHLH3, bHLH13, bHLH14 and bHLH17 transcription factors as novel transcription repressors of JA signaling. The coordinated regulation of JA-mediated plant defense and development by transcription activators and repressors would improve the survival of plants in their natural habitat and adaptation to the frequently fluctuating environment.

## Introduction

Plant hormones are essential for the regulation of plant growth, differentiation, development, reproduction, and survival [1]–[3]. Jasmonates (JAs), a class of cyclic fatty acid-derived plant hormones originating from plastid membrane α-linolenic acid [4], [5], regulate diverse aspects of plant developmental processes [6], such as seedling growth [7], root development [8]–[10], plant fertility [11]–[13], trichome initiation [14], [15], pigment formation [14], [16], [17], and senescence [18]. It is also well established that jasmonates act as key defense signals in regulation of various abiotic and biotic stress such as mechanic wounding [19], [20], arthropod herbivores and necrotrophic pathogens [21]–[24] and drought [25], [26].

In response to external environmental signals and internal developmental cues, plants generate and perceive jasmonates (JA) signals to induce degradation of JASMONATE ZIM-Domain (JAZ) proteins [20], [27]–[33]. As consequence of JAZs degradation, JAZ-targeted transcription factors will be relieved to subsequently regulate their downstream signal cascades and modulate respective JA responses.

Current studies have identified several key transcription factors as direct targets of JAZ proteins [34]–[38]. The bHLH subgroup IIIe transcription factors (MYC2, MYC3 and MYC4) are targets of JAZs and play important roles in regulation of plant defense and developmental processes [39]–[41]. The R2R3-MYB transcription factors (MYB21, MYB24 and MYB57), and the transcription complexes WD-repeat/bHLH (TT8, GL3 or EGL3)/MYB (MYB75 or GL1) interact with JAZs to regulate JA-mediated male fertility, anthocyanin accumulation and trichome initiation, respectively [12], [14], [42]. Current research so far has not identified JAZ-targeted transcription repressors in JA pathway.

In this study, we identified the bHLH subgroup IIId transcription factors (bHLH3, bHLH13, bHLH14 and bHLH17) as new targets of JAZ proteins. These bHLH subgroup IIId transcription factors function redundantly to negatively regulate JA-mediated plant defense and development. Furthermore, these bHLH factors act as transcriptional repressors to suppress JA responses, which antagonize the previously reported transcription activators (such as MYC2 and the WD-repeat/TT8/MYB75 complex) through binding to their downstream target sequences. Coordinated regulation of JA responses by transcription repressors and activators would benefit plants for adaptation to their frequently changing environment.

## Results

### JAZ Proteins Interact with the bHLH Subgroup IIId Transcription Factors bHLH3, bHLH13, bHLH14 and bHLH17

**Figure 1:**
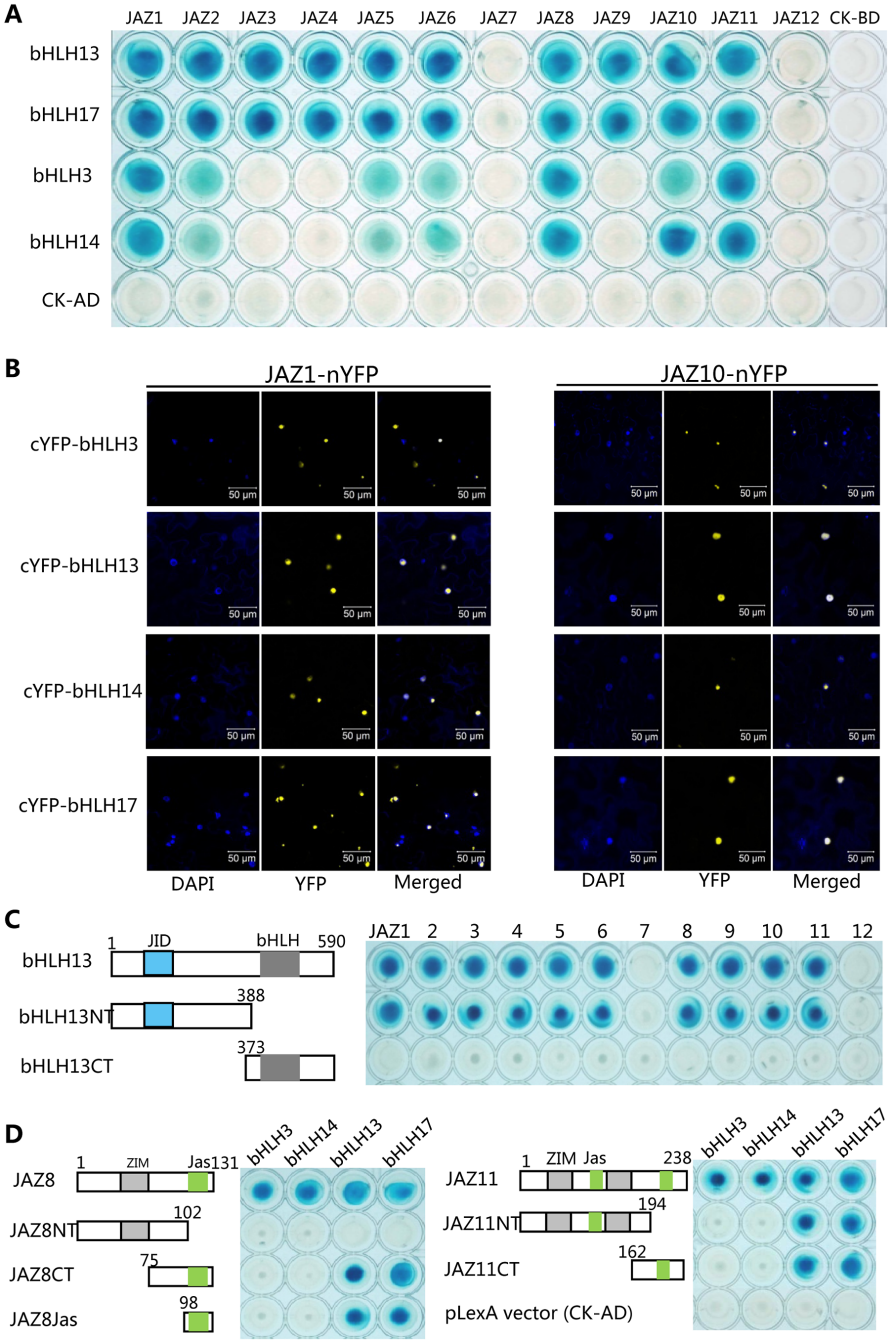
JAZ proteins interact with bHLH3, bHLH13, bHLH14 and bHLH17. (A) Yeast two-hybrid (Y2H) assay to detect interactions of JAZs with bHLH3, bHLH13, bHLH14 and bHLH17 factors. Twelve *Arabidopsis* JAZs were fused with the LexA DNA binding domain (BD) in pLexA respectively. The bHLH3, bHLH13, bHLH14 and bHLH17 were fused with the activation domain (AD) in pB42AD respectively. Interactions (represented by blue color) were assessed on 2% Gal/1% raffinose/SD/-Ura/-His/-Trp/-Leu/X-β-Gal medium. (B) Bimolecular fluorescence complementation (BiFC) assay to detect interactions of JAZ1 and JAZ10 (fused with nYFP) with bHLH3, bHLH13, bHLH14 and bHLH17 (fused with cYFP). Construct pairs were coexpressed in leaves of *N. benthamiana*. YFP fluorescence was detected 50 hours after infiltration. The nuclei were indicated by DAPI (4,6-diamidino-2-phenylindole dihydrochloride) staining. (C) Y2H assay to test interactions of bHLH13 domain constructs with twelve JAZs. The schematic diagram shows the bHLH13 domain constructs. The conserved JID domain and bHLH domain were shown with blue and gray box respectively. The numbers indicate the positions of amino acid. Different bHLH13 domains were fused with AD in pB42AD, and JAZs were fused with BD in pLexA. (D) Y2H assay to test interactions between different domains of JAZ8 and JAZ11 with bHLH3, bHLH13, bHLH14 and bHLH17 respectively. The schematic diagram shows the JAZ8 and JAZ11 domain constructs. The conserved ZIM and Jas domains are indicated by gray and green boxes respectively. Different domains of JAZ8 and JAZ11 were fused with BD, and these bHLH factors were fused with AD individually.

To understand molecular basis of jasmonate action, we exhaustedly screened the *Arabidopsis thaliana* cDNA library using JAZ proteins (JAZ1 and JAZ8) as bait in the yeast two-hybrid (Y2H) system, and identified several transcription factors [12], [14]. We found that a bHLH transcription factor bHLH13 (AT1G01260) also interacted with various JAZ proteins (Figure 1A).

Phylogenetic analysis showed that bHLH13, together with bHLH3 (AT4G16430), bHLH14 (AT4G00870) and bHLH17 (AT2G46510/AtAIB), belongs to the subgroup IIId of the *Arabidopsis* bHLH family [43]–[45]. We further found that, in the yeast two-hybrid (Y2H) assays, all the bHLH subgroup IIId factors interacted with various JAZ proteins (Figure 1A).

To verify the interactions between JAZs and the bHLH3, bHLH13, bHLH14 or bHLH17 in planta, we performed bimolecular fluorescence complementation (BiFC) assays in leaves of *Nicotiana benthamiana*. As shown in Figure 1B, the strong signals of yellow fluorescent protein (YFP) in nucleus of *N. benthamiana* leaves were reconstructed by coexpression of JAZ1-nYFP (the JAZ1 fused with N-terminal fragment of YFP) with cYFP-bHLH3, cYFP-bHLH13, cYFP-bHLH14 and cYFP-bHLH17, respectively. Strong YFP signals were also detected when JAZ10 was coexpressed with these transcription factors in the BiFC assays (Figure 1B). These results suggested that these bHLH subgroup IIId factors interact with JAZs in planta.

To investigate which domain of these bHLH factors is responsible for the interaction with JAZ proteins, we representatively divided the bHLH13 into the N-terminal fragment (bHLH13NT) containing the JAZ-Interaction-Domain (JID) [40], and the C-terminal part (bHLH13CT) (Figure 1C). As shown in Figure 1C, bHLH13NT, but not bHLH13CT exhibited interactions with various JAZ proteins. Consistent with the previous speculation [40], these results suggest that the N-terminal fragment (JID domain) of the bHLH13 factor is responsible for the interactions with JAZ proteins.

To further examine which domain of JAZ proteins is critical for interactions with the bHLH subgroup IIId factors, we divided JAZ8 into the N-terminal fragment (JAZ8NT), the C-terminal part (JAZ8CT), and the Jas domain only (JAZ8Jas) (Figure 1D). Our results suggested that the Jas domain in JAZ8 is required for interactions with bHLH13 and bHLH17 (Figure 1D). Interestingly, bHLH3 and bHLH14 were able to interact with JAZ8, but not with the truncated fragments of JAZ8 (JAZ8NT, JAZ8CT and JAZ8Jas) (Figure 1D), suggesting that the entire JAZ8 is required for interactions with bHLH3 and bHLH14. Similarly, we found that the entire JAZ11 is required for interactions with bHLH3 and bHLH14, while either of Jas domains in JAZ11 is sufficient for interactions with bHLH13 and bHLH17 (Figure 1D).

### The bHLH3, bHLH13, bHLH14 and bHLH17 Function Redundantly to Negatively Regulate JA Responses

**Figure 2:**
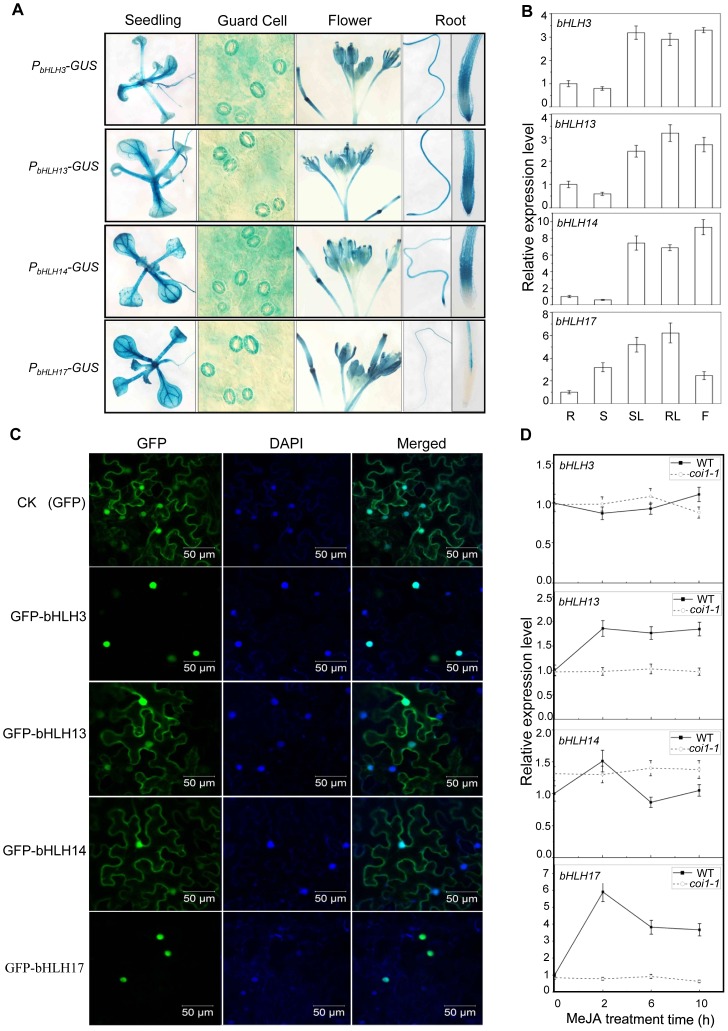
Expression patterns and subcellular localizations of bHLH3, bHLH13, bHLH14 and bHLH17. (A) *GUS* reporter gene was fused with the promoters of the four bHLH factors respectively to generate *Arabidopsis* transgenic plants (*P_bHLH3_*-*GUS*, *P_bHLH13_*-*GUS*, *P_bHLH14_*-*GUS* and *P_bHLH17_*-*GUS*). Histochemical GUS activity was detected in various tissues of transgenic seedlings. (B) Quantitative real-time PCR analysis of relative expression levels of *bHLH3*, *bHLH13*, *bHLH14* and *bHLH17* in root (R), stem (S), rosette leaf (RL), stem leaf (SL) and flower (F). *ACTIN8* was used as the internal control. Error bars represent SE (n = 3). (C) Subcellular localization of bHLH3, bHLH13, bHLH14 and bHLH17 in epidermal cells of *N. benthamiana* leaves. Constructs indicated on the left were infiltrated in leaves of *N. benthamiana*. GFP fluorescence was detected 50 hours after infiltration. The nuclei were indicated by DAPI staining. (D) Quantitative real-time PCR analysis of *bHLH3*, *bHLH13*, *bHLH14* and *bHLH17* in 11-day-old WT and *coi1-1* seedlings treated with 100 µM methyl-jasmonate (MeJA) for 0, 2, 6, and 10 hours. *ACTIN8* was used as the internal control. Error bars represent SE (n = 3).

To examine expression patterns of *bHLH3*, *bHLH13*, *bHLH14* and *bHLH17* in plant tissues, we generated *Arabidopsis* plants transgenic for the GUS reporter driven by endogenous promoter of each *bHLH* factor. Histochemical staining of the GUS activity demonstrated that all these *bHLH* factors are expressed in various plant tissues (Figure 2A), which is consistent with quantitative real-time PCR analysis (Figure 2B) and the public available data (www.bar.utoronto.ca) [43]. Interestingly, the *COI1*-dependent JA-induced gene expression was observed for *bHLH13* and *bHLH17*, but not for *bHLH3* and *bHLH14* (Figure 2D). Further examination of subcellular localization indicated that bHLH3 and bHLH17 were nucleus-localized (Figure 2C), whereas bHLH13 and bHLH14 were localized in both nucleus and cytoplasm (Figure 2C).

**Figure 3:**
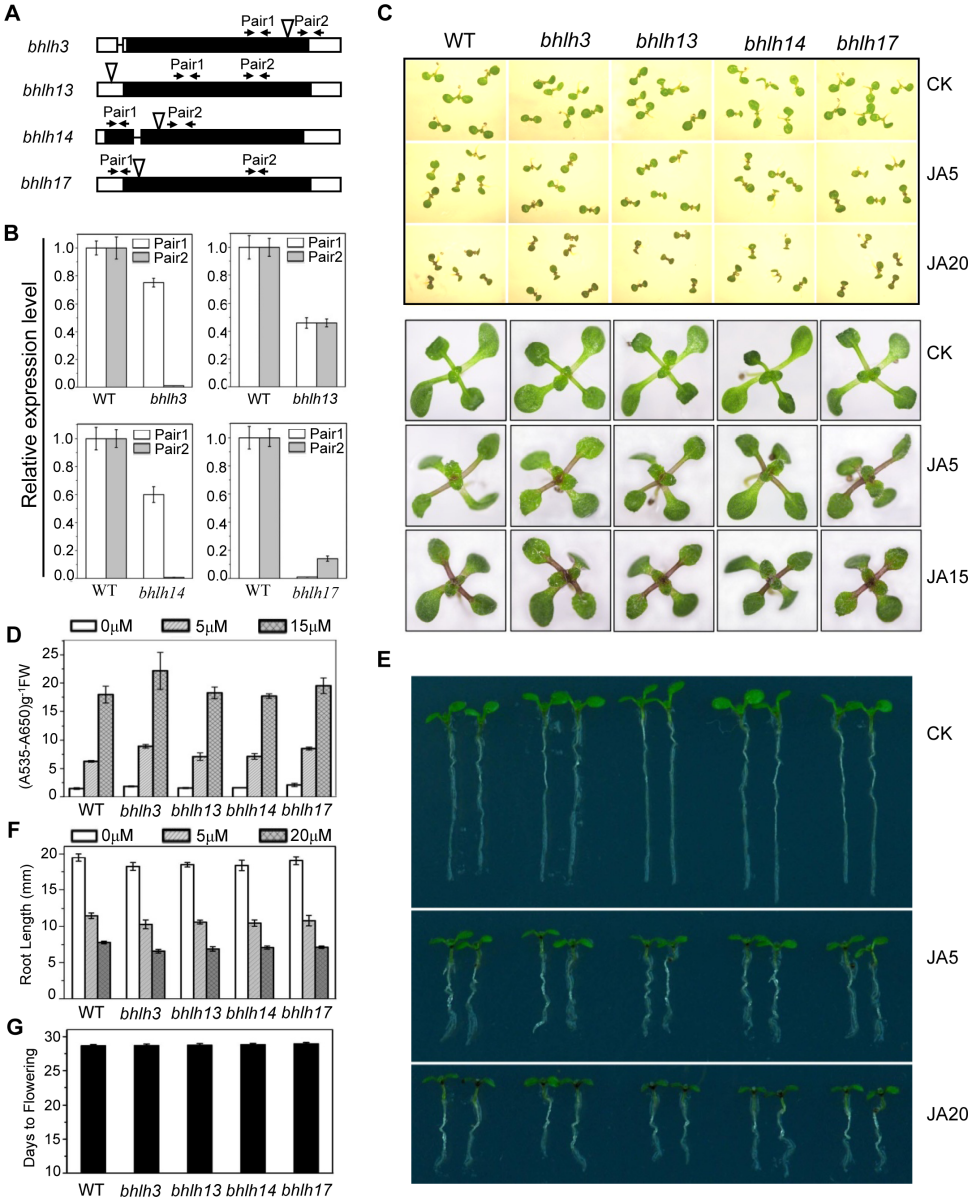
JA responses were not obviously altered in the *bhlh3*, *bhlh13*, *bhlh14* and *bhlh17* single mutants. (A) Schematic diagrams of T-DNA insertion sites in *bHLH3*, *bHLH13*, *bHLH14* and *bHLH17*. White box, UTR; black box, exon; black line, intron; white triangle, T-DNA insertion site. Pair1 and Pair2, indicated by arrows, are the primer pairs for analyzing gene expression in (B). (B) Quantitative real-time PCR analysis of *bHLH3*, *bHLH13*, *bHLH14* and *bHLH17* in the respective T-DNA insertion mutants using primer pairs indicated by arrow pairs in (A). *ACTIN8* was used as the internal control. (C) Seedling phenotypes of 7-day-old (upper panel) and 11-day-old (bottom panel) Col-0 wild type (WT), *bhlh3*, *bhlh13*, *bhlh14* and *bhlh17* grown on MS medium supplied without (CK) or with indicated concentrations (µM) of MeJA. (D) Anthocyanin contents of the 11-day-old seedlings in WT, *bhlh3*, *bhlh13*, *bhlh14* and *bhlh17* single mutants grown on MS medium containing indicated concentrations of MeJA. FW, fresh weight. Error bars represent SE (n = 3). (E) Root phenotypes of 7-day-old seedlings of WT and single mutants of *bhlh3*, *bhlh13*, *bhlh14* and *bhlh17* grown on MS medium supplied without (CK) or with indicated concentrations (µM) of MeJA. (F) Root length of 11-day-old seedlings grown on MS medium containing indicated concentrations of MeJA. Error bars represent SE (n = 15). (G) Flowering time of WT, *bhlh3*, *bhlh13*, *bhlh14* and *bhlh17* single mutants. Data shown are the means from 24 plants. Error bars represent SE.

To investigate the function of the bHLH subgroup IIId factors, we identified *Arabidopsis* mutants for *bHLH3*, *bHLH13*, *bHLH14* and *bHLH17* with the T-DNA insertion into the exon (for *bHLH3*, *bHLH14* and *bHLH17*) or the 5′UTR (for *bHLH13*) (Figure 3A). Quantitative real-time PCR analysis showed that the expression of the full length bHLH subgroup IIId gene was abolished (for *bHLH3*, *bHLH14* and *bHLH17*) or obviously reduced (for *bHLH13*) in their respective mutants (Figures 3A and 3B). Observation of typical JA-regulated responses, including plant fertility, JA-inhibitory root growth and JA-induced anthocyanin accumulation, showed that no obvious differences were detected among wild-type, *bhlh3*, *bhlh13*, *bhlh14* and *bhlh17* single mutants (Figure 3, and data not shown).

**Figure 4:**
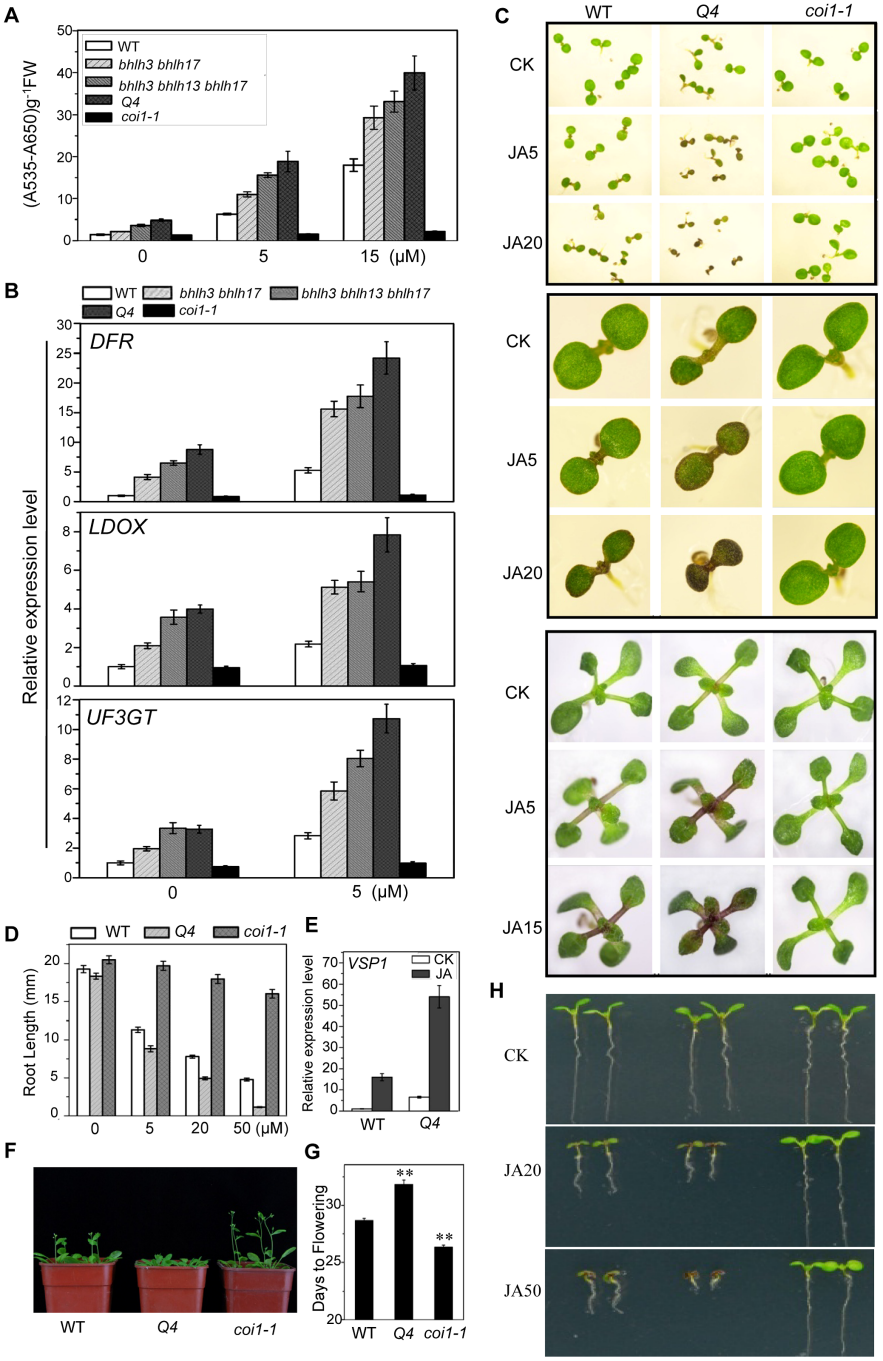
The *bhlh3 bhlh13 bhlh14 bhlh17* quadruple mutant exhibited enhanced JA responses. (A) Anthocyanin contents in the 11-day-old seedlings of WT, *bhlh3 bhlh17*, *bhlh3 bhlh13 bhlh17*, *bhlh3 bhlh13 bhlh14 bhlh17* (*Q4*) and *coi1-1* grown on MS medium containing indicated concentrations of MeJA. FW, fresh weight. Error bars represent SE (n = 3). (B) Quantitative real-time PCR analysis of *DFR*, *LDOX* and *UF3GT* expression levels in 11-day-old seedlings of indicated plants grown on MS medium supplied with 0 or 5 µM MeJA. *ACTIN8* was used as the internal control. (C) Phenotypes of 7-day-old (the upper panel, the middle panel that showed enlarged seedlings) and 11-day-old seedlings (the bottom panel) of WT, the quadruple mutant (*Q4*) and *coi1-1* grown on MS medium supplied without (CK) or with indicated concentrations (µM) of MeJA. (D) Root length of 11-day-old seedlings grown on MS medium containing indicated concentrations of MeJA. Error bars represent SE (n = 15). Asterisks denote Student's t-test significance compared with WT plants: **, P<0.01. (E) Quantitative real-time PCR analysis of *VSP1* expression level in WT, *Q4* and *coi1-1* seedlings grown for 11 days on MS medium supplied without (CK) or with 5 µM MeJA (JA). *ACTIN8* was used as the internal control. (F) Flowering phenotypes of five-week-old WT, *Q4* and *coi1-1* plants grown in a long-day growth chamber (16L/8D, 23°C). (G) Flowering time of WT, *Q4* and *coi1-1*. Data shown are the means from 24 plants. Error bars represent SE. Asterisks denote Student's t-test significance compared with WT plants: **, P<0.01. (H) Root phenotypes of 7-day-old WT, *Q4* and *coi1-1* seedlings grown on MS medium supplied without (CK) or with indicated concentrations (µM) of MeJA.

As *bHLH3*, *bHLH13*, *bHLH14* and *bHLH17* display high similarity at the amino acid level and belong to the same subgroup IIId of the *Arabidopsis* bHLH family [44], we further generated double, triple and quadruple mutants for the bHLH subgroup IIId factors, through genetic cross among the *bhlh3*, *bhlh13*, *bhlh14* and *bhlh17* mutants, to investigate whether these factors function redundantly in regulation of JA responses. Interestingly, we found that the anthocyanin accumulation was gradually increased in the double mutant *bhlh3 bhlh17*, the triple mutant *bhlh3 bhlh13 bhlh17*, *bhlh3 bhlh13 bhlh14*, *bhlh3 bhlh14 bhlh17*, *bhlh13 bhlh14 bhlh17*, and the quadruple mutant *bhlh3 bhlh13 bhlh14 bhlh17* in response to MeJA treatment (Figures 4A, 4C, and S1). Consistent with the tendency of JA-induced anthocyanin accumulation (Figures 4A and 4C), MeJA-induced expression of the anthocyanin biosynthetic genes, including *DFR*, *LDOX* and *UF3GT* [46], was gradually increased in the double, triple and quadruple mutants (Figure 4B).

The quadruple mutant *bhlh3 bhlh13 bhlh14 bhlh17* exhibited enhanced JA responses. They displayed obvious increase in JA-induced anthocyanin biosynthesis compared with wild-type control (Figures 4A and 4C). The JA-inhibitory root growth analysis also showed that the quadruple mutant was more sensitive to JA inhibition of root growth (Figures 4D and 4H). Observation of flowering time showed that the quadruple mutant also exhibited enhanced JA response: the quadruple mutant exhibited late flowering phenotype whereas the *coi1*-*1* mutant plant flowered early (Figures 4F and 4G). Consistent with the enhanced JA-responses, expression of JA-inducible marker gene *VSP1* [7] was significantly increased in the quadruple mutant (Figure 4E).

Taken together (Figures 3 and 4), mutations in *bHLH3*, *bHLH13*, *bHLH14* and *bHLH17* caused enhanced JA responses, demonstrating that these bHLH subgroup IIId factors function redundantly to negatively regulate JA responses.

### The Quadruple Mutant *bhlh3 bhlh13 bhlh14 bhlh17* Exhibited Enhanced JA-regulated Plant Defense

**Figure 5:**
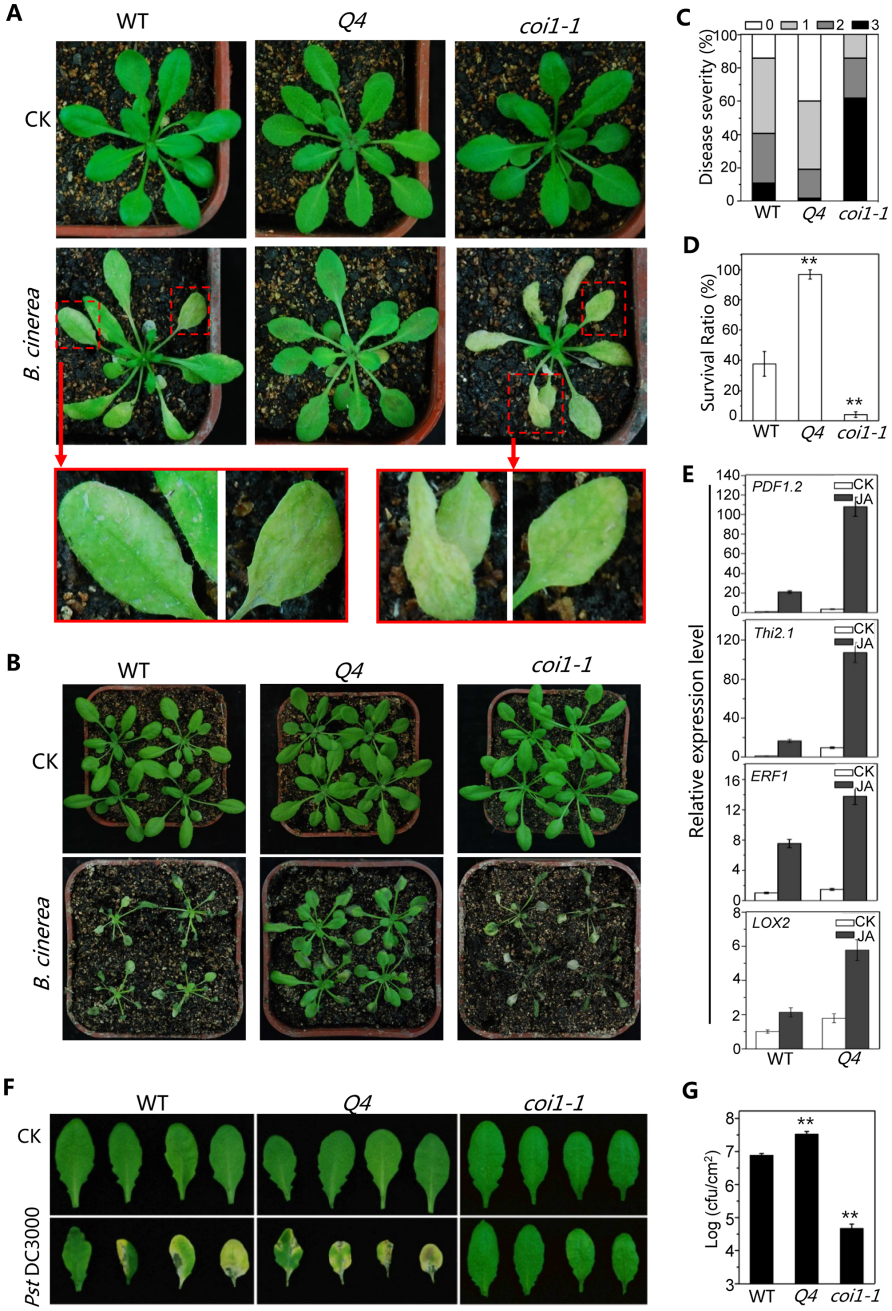
The bHLH subgroup IIId factors negatively regulate JA-mediated plant defense. (A) Disease symptoms of WT, the quadruple mutant (*Q4*) and the *coi1-1* mutant five days after spray inoculation with *Botrytis cinerea* or mock (CK) in the growth condition with ∼40% humidity. The enlarged leaves indicate obvious disease symptoms in WT, and severe disease symptoms *in coi1-1*. (B) Disease symptoms of WT, the quadruple mutant (*Q4*) and *coi1-1* mutant seven days after spray inoculation with *B. cinerea* or mock (CK) in the growth condition with high humidity (>90%). (C) Disease severity of the plants in (A) seven days after spray inoculation with *B. cinerea*. Disease rating was represented as no visible (0, white), weak (1, light grey), severe symptoms (2, grey), and completely dead plants (3, dark). Experiments were repeated three times with similar results. (D) Survival ratio of the plants in (B) nine days after spray inoculation. Error bars represents SE. Asterisks denote Student's t-test significance compared with WT plants: **, P<0.01. (E) Quantitative real-time PCR analysis of expression levels of *PDF1.2*, *Thi2.1*, *ERF1* and *LOX2* in WT and *Q4* germinated and grown on MS medium supplied without (CK) or with 5 µM MeJA (JA) for 11 days. *ACTIN8* was used as the internal control. (F) Disease symptoms on leaves of WT, *Q4* and *coi1-1* mutant three days after spray inoculation with *Pseudomonas syringae* pv. *tomato* (*Pst*) DC3000. (G) Growth of *Pst* DC3000 on WT, *Q4* and *coi1-1* mutant plants three days after spray inoculation as in (F). Bacterial counts are expressed as log (cfu/cm^2^). Error bars represent SE. The results are representative of three independent biological experiments. Asterisks represent Student's t-test significance compared with WT plants: **, P<0.01.

*Botrytis cinerea*, a necrotrophic fungus that causes gray mold disease in many plant species [47], induced severe wilting and high mortality in the *Arabidopsis* mutants *coi1-1* [8], [48] or *aos1* [49], [50]. To investigate potential role for *bHLH3*, *bHLH13*, *bHLH14* and *bHLH17* in plant defense, we sprayed the *B. cinerea* spore suspension onto the quadruple mutant, wild-type and *coi1-1* plants. As shown in Figure 5A and 5B, the quadruple mutant plants exhibited increased resistance against *B. cinerea* compared with wild type, whereas *coi1-1* mutant plants displayed severe disease symptom, as revealed by disease severity and plant survival rate (Figures 5C and 5D). Consistent with the increased defense response, the PLANT-DEFENSIN gene *PDF1.2* [51], antifungal gene *THI2.1* [52], defense gene *ERF1* [53] and wound-inducible gene *LOX2* [54] were highly induced in the quadruple mutant compared with wild type when treated with MeJA (Figure 5E).

Previous studies showed that JA induces plant susceptibility to the bacterium strain *Pst* DC3000 of *Pseudomonas syringae* pv. *Tomato*. The *coi1-1* mutant is more resistant to the *Pst* DC3000 inoculation [8], [55], [56] (Figures 5F and 5G). The quadruple mutant exhibited enhanced JA response in the *Pst* DC3000 inoculation assay: the quadruple mutant was more susceptible to the *Pst* DC3000 infection, whereas *coi1-1* was resistant (Figures 5F and 5G).

**Figure 6:**
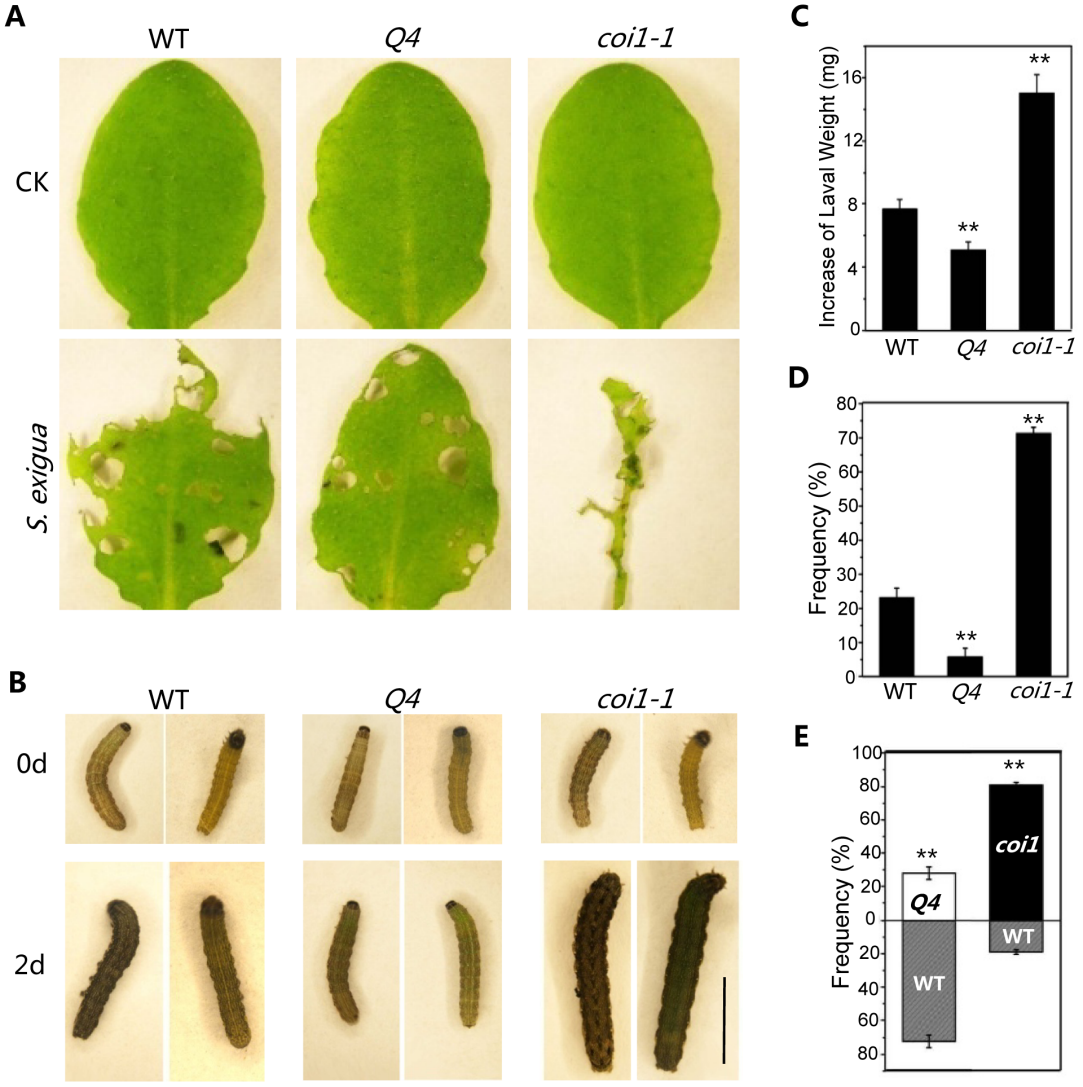
The *bhlh3 bhlh13 bhlh14 bhlh17* quadruple mutant exhibited enhanced JA-mediated plant defense against insect. (A) Representative rosette leaves of wild-type (WT), the quadruple mutant *bhlh3 bhlh13 bhlh14 bhlh17* (*Q4*), and *coi1-1* after feeding without (CK) or with *S. exigua*. (B) Representative *S. exigua* larvae before feeding (0 d) or two days after feeding (2 d) with rosette leaves WT, *Q4* and *coi1-1*. The scale bar represents 5 mm. (C) Increase of *S. exigua* larval weight two days after feeding with rosette leaves of WT, *Q4* and *coi1-1*. Error bars represent SE. Asterisks represent Student's t-test significance compared with WT plants: **, P<0.01. (D) The preference of *S. exigua* larvae in the *three*-*choice test* among WT, *Q4* and *coi1-1*. Error bars represent SE. Asterisks represent Student's t-test significance compared with WT plants: **, P<0.01. (E) The preference of *S. exigua* larvae in the *two*-*choice test* between WT and *Q4*, or between WT and *coi1-1*. Error bars represent SE. Asterisks represent Student's t-test significance compared with WT plants: **, P<0.01.

To test whether the bHLH subgroup IIId factors regulate JA-mediated plant defense against insects, mature rosette leaves of wild-type, *coi1-1*, and the quadruple mutant were fed to *Spodoptera exigua*, a globally-significant agricultural pest with a broad host range [21]. We found that *S. exigua* larvae consumed the majority of the *coi1-1* leaves (Figure 6A) and grew rapidly (Figures 6 B and C). However, the quadruple mutant leaves inhibited the growth of *S. exigua* larvae and showed reduced consumption by *S. exigua* (Figures 6 A–C). When insects were given the choice of selecting among wild type, *coi1-1* and the quadruple mutant in the *three*-*choice test*, the quadruple mutant attracted fewer *S. exigua* larvae (∼6%), while wild type and *coi1-1* accommodated the majority of *S. exigua* larvae (23% for wild type, 71% for *coi1-1*) (Figure 6D). In the *two-choice test*, the quadruple mutant attracted less larvae than wild type (∼28% for the quadruple mutant, ∼72% for wild type), while the wild-type attracted less larvae than *coi1-1* (19% for wild type, ∼81% for *coi1-1*) (Figure 6E). These results suggested that the quadruple mutant displays enhanced JA-regulated plant defense against insect.

In summary, we demonstrated that the bHLH subgroup IIId factors (bHLH3, bHLH13, bHLH14 and bHLH17) function as novel negative regulators of JA-mediated plant defense.

### Overexpression of the Subgroup IIId bHLH Factor Caused Reduced JA Responses

**Figure 7:**
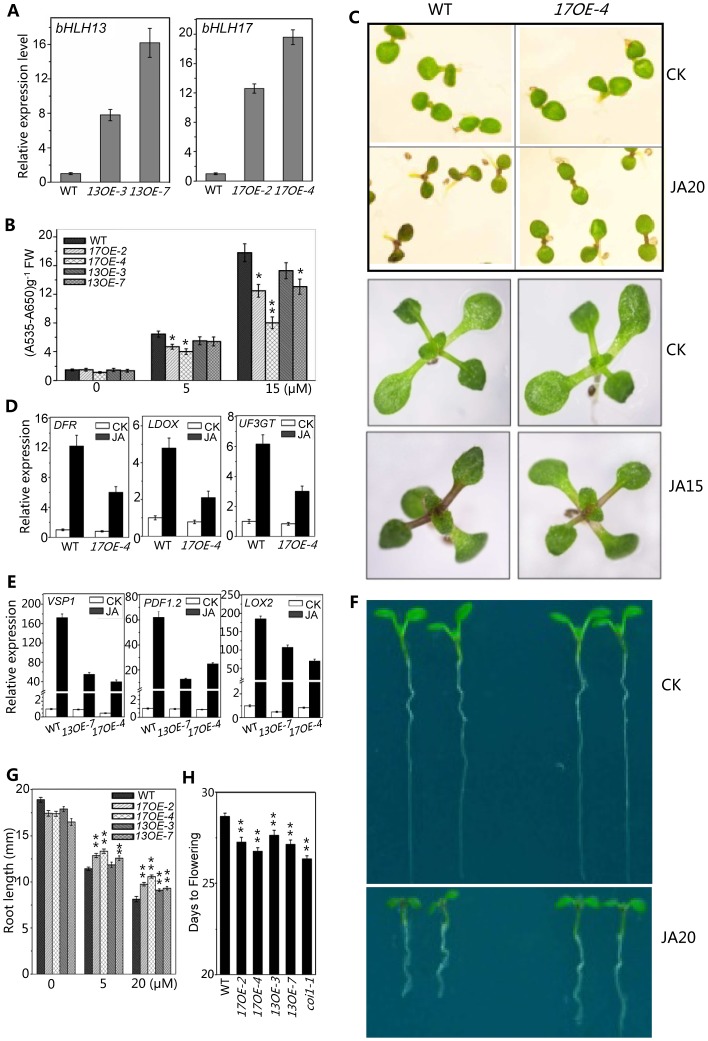
Overexpression of *bHLH 13* or *bHLH 17* attenuated JA responses. (A) Quantitative real-time PCR analysis of expression levels of *bHLH13* and *bHLH17* in 11-day-old seedlings of WT and transgenic plants overexpressing *bHLH13* or *bHLH17*. Two individual transgenic lines for *bHLH13* (*13OE-3* and *13OE-7*) and *bHLH17* (*17OE-2* and *17OE-4*) were representatively used in analysis. *ACTIN8* was used as the internal control. (B) Anthocyanin contents of the indicated 11-day-old seedlings grown on MS medium supplied with indicated concentrations of MeJA. FW, fresh weight. Error bars represent SE (n = 3). Asterisks represent Student's t-test significance compared with WT plants: *, P<0.05; **, P<0.01. (C) Phenotypes of 7-day-old (upper panel) and 11-day-old (bottom panel) seedlings of WT and *17OE-4* grown on MS medium supplied without (CK) or with indicated concentrations (µM) of MeJA. (D) Quantitative real-time PCR analysis of expression levels of *DFR*, *LDOX* and *UF3GT* in 11-day-old seedlings of WT and *17OE-4* grown on MS medium supplied without (CK) or with 15 µM MeJA (JA). *ACTIN8* was used as the internal control. (E) Quantitative real-time PCR analysis of expression levels of *VSP1*, *PDF1.2* and *LOX2* in 11-day-old seedlings of WT, *13OE-7* and *17OE-4* treated with mock (CK) or with 100 µM MeJA (JA) for 6 hours. *ACTIN8* was used as the internal control. (F) Root phenotypes of 7-day-old WT and *17OE-4* seedlings grown on MS medium supplied without (CK) or with 20 µM MeJA. (G) Root length of 11-day-old WT, *bHLH13* and *bHLH17* overexpression transgenic seedlings grown on MS medium containing indicated concentrations of MeJA. Error bars represent SE (n = 15). Asterisks represent Student's t-test significance compared with WT plants: **, P<0.01. (H) Flowering time of WT, *bHLH13* and *bHLH17* overexpression transgenic lines. Data shown are the means from 24 plants. Error bars represent SE. Asterisks represent Student's t-test significance compared with WT plants: **, P<0.01.

Having demonstrated that the quadruple mutant exhibited enhanced JA responses (Figures 4–6), we further investigated whether overexpression of the bHLH subgroup IIId factors would lead to reduction in JA responses. As shown in Figure 7, the *bHLH17* overexpression lines, *17OE-2* and *17OE-4* with highly expressed *bHLH17* transcripts (Figure 7A), exhibited reduced JA responses compared with wild type, as indicated by reduction in JA-induced anthocyanin accumulation (Figures 7B and 7C), JA-inducible anthocyanin biosynthetic gene expression (Figure 7D), and JA-inhibitory root growth (Figures 7F and 7G). Similar to the *bHLH17* overexpression plants, the *bHLH13* overexpression lines (*13OE-3* and *13OE-7*) also exhibited reduced JA responses (Figure 7). These overexpression lines also exhibited the *coi1*-like phenotype to flower early (Figure 7H). Consistent with the decreased JA responses, JA-inducible gene expression of *VSP1*, *LOX2*, and *PDF1.2* was reduced in the *bHLH13* or *bHLH17* overexpression lines (Figure 7E).

Taken together with data of the genetic and physiological analysis on the quadruple mutant and the overexpression transgenic lines (Figures 4–7), we demonstrated that the bHLH3, bHLH13, bHLH14 and bHLH17 function as novel negative regulators of JA responses.

### The bHLH Subgroup IIId Factors Act as Transcription Repressors

**Figure 8:**
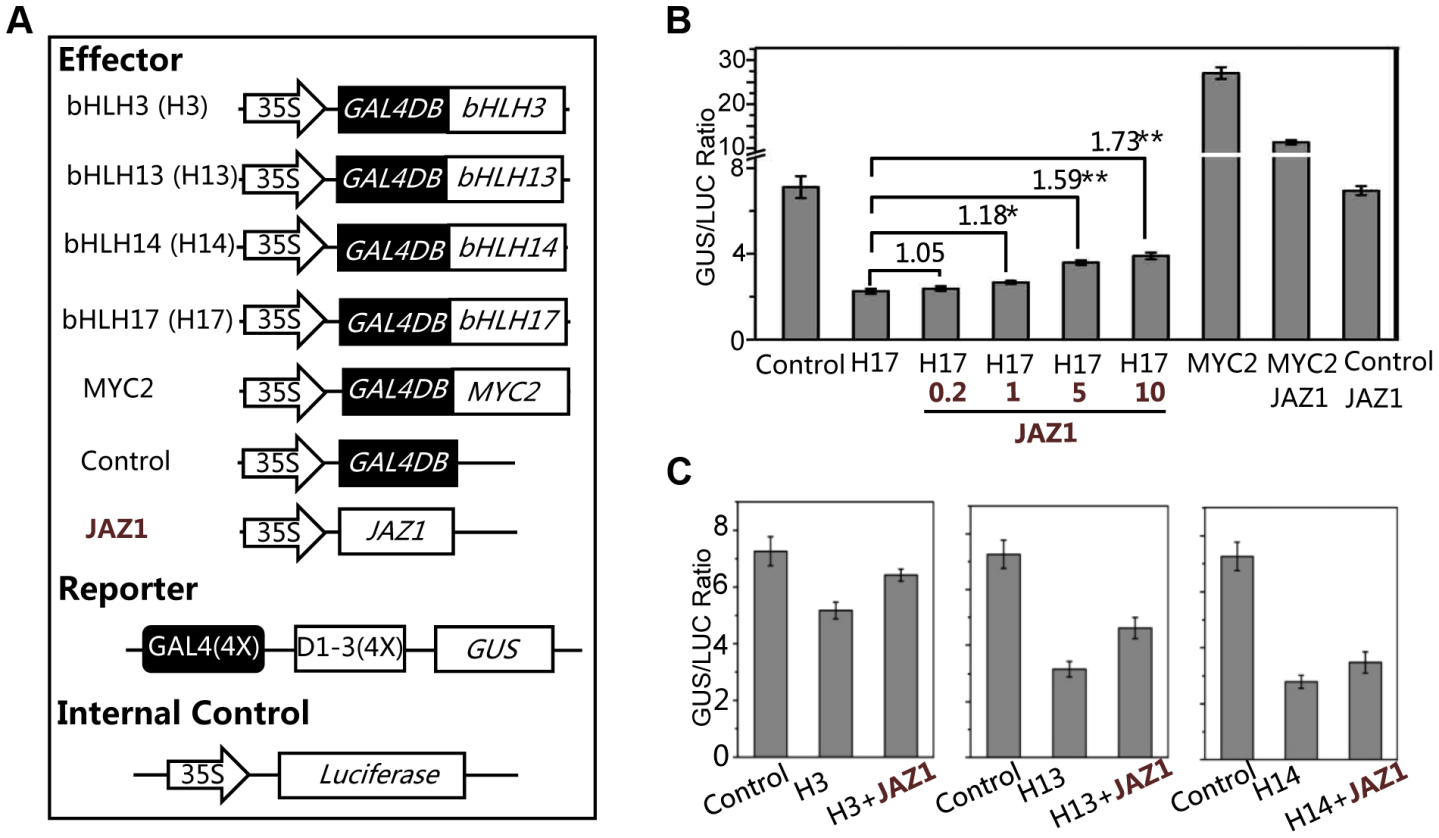
bHLH3, bHLH13, bHLH14 and bHLH17 act as transcription repressors. (A) The schematic diagram shows the constructs used in the transient expression assays. (B) Transient expression assays show that the bHLH17 (indicated by H17) acts as a transcription repressor, while JAZ1 attenuates the bHLH17 repression function in a dosage-dependent manner. The MYC2 functions as a transcription activator, while JAZ1 represses the MYC2 activation activity. The GUS reporter and the internal control luciferase were cotransformed with the Control, bHLH17 (H17) and MYC2 effectors. The 0.2, 1, 5 or 10 µg JAZ1 plasmid DNA were respectively used for co-transformation with bHLH17 (H17). 10 µg JAZ1 plasmid DNA was used for co-transformation with MYC2 and Control. When JAZ1 was used for co-transformation with the Control vector only, the GUS activity was not affected, which is consistent with the previous observations that the negative regulator JAZ1 is not a transcription factor and cannot repress the promoter sequences (JAZ1 interacts with and represses its targeted proteins/transcription factors). Numbers on the brackets indicate the relative values of the GUS/LUC ratio of bHLH17 with JAZ1 to that of bHLH17. The GUS/LUC ratio represents the GUS activity relative to the internal control LUC. Error bars represent SE (n = 3). Asterisks represent Student's t-test significance between pairs indicated with brackets (*, P<0.05; **, P<0.01). (C) Transient expression assays show that bHLH3, bHLH13 and bHLH14 act as transcription repressors and their repression function can be attenuated by JAZ1. The GUS reporter and the luciferase (LUC) internal control were cotransformed with the indicated constructs (10 µg for each construct). Error bars represent SE (n = 3).

Having shown that the bHLH subgroup IIId factors (bHLH3, bHLH13, bHLH14 and bHLH17) negatively regulat JA responses, we examined whether these bHLH subgroup IIId factors function as transcriptional repressors using the GAL4-DNA- binding-domain (GAL4DB) and its binding sites (GAL4(4X)-D1-3(4X)-GUS)-based protoplast transient expression system [57], [58]. We found that the MYC2 functions as a transcription activator whereas JAZ1 acts as a negative regulator to inhibit the MYC2 activation activity in this transient expression system (Figure 8A and 8B). In contrast, bHLH17 functions as a transcription repressor whereas JAZ1 acts as a negative regulator to inhibit the bHLH17 repression function: expression of GAL4DB-fused bHLH17 obviously repressed the activity of the GUS reporter (Figure 8A and 8B); furthermore, coexpression of JAZ1 inhibited the bHLH17 repression function and rescued the bHLH17-repressed GUS activity in a dosage-dependent manner (Figure 8B).

Similarly, we observed that GAL4DB-fused bHLH3, bHLH13 or bHLH14 repressed the activity of GUS reporter, and that their repression function could be partially inhibited by JAZ1 (Figure 8C). These results collectively suggest that these bHLH subgroup IIId factors act as transcriptional repressors, and that JAZ proteins interact with these transcription factors to attenuate their repression function.

### The bHLH Subgroup IIId Factors Antagonize Transcription Activators in JA Pathway

**Figure 9:**
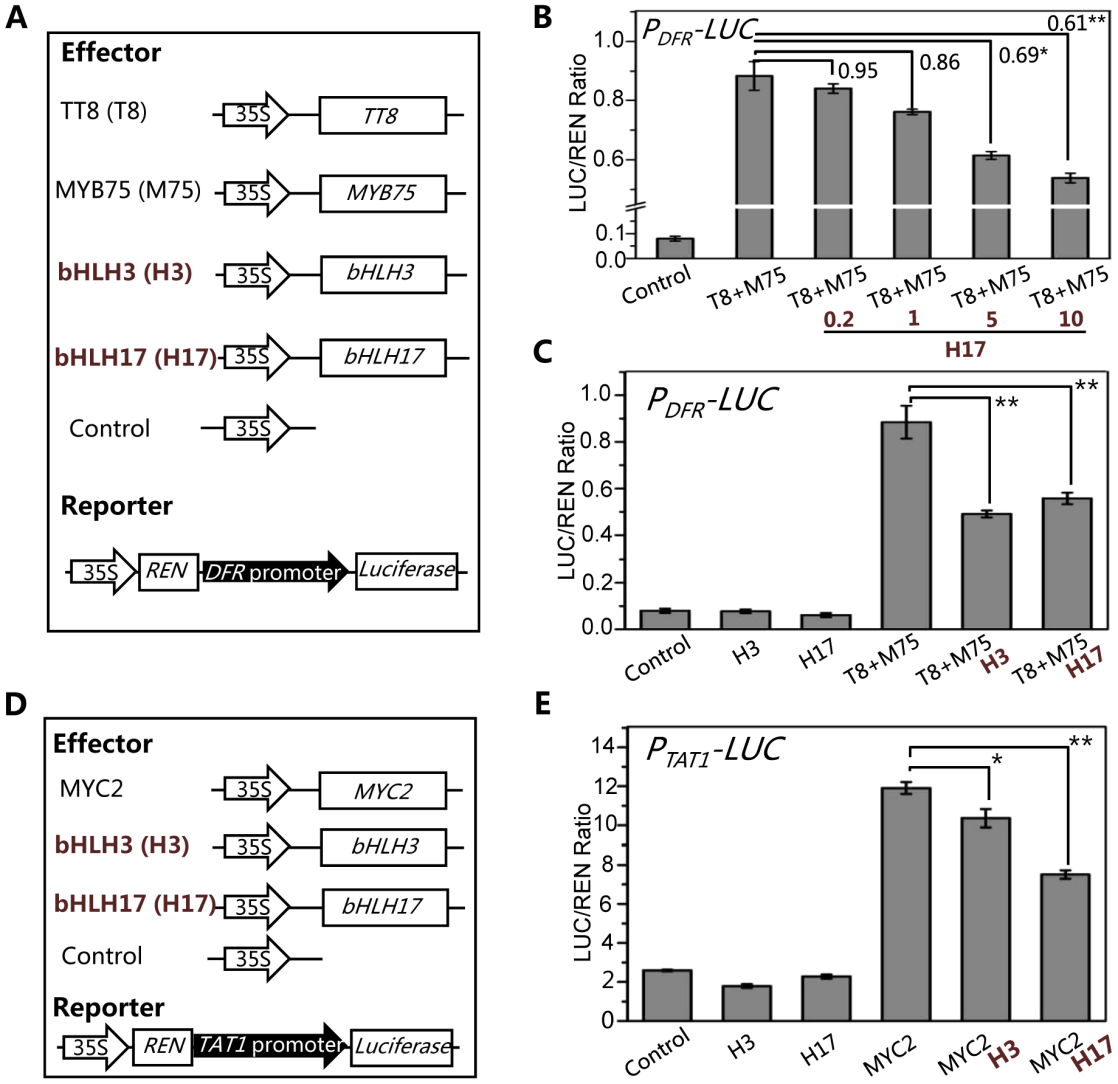
The bHLH3 and bHLH17 antagonize TT8/MYB75 and MYC2 to negatively regulate their downstream target genes. (A) The schematic diagram shows the constructs used in the transient transcriptional activity assays of (B) and (C). (B) Transient transcriptional activity assays show that activation of *DFR* promoter by TT8/MYB75 is repressed by bHLH17 in dosage-dependent manner. The *P_DFR_-LUC* reporter was cotransformed with the indicated constructs. The 0.2, 1, 5 or 10 µg bHLH17 (H17) plasmid DNA were respectively used for co-transformation with TT8 (T8) and MYB75 (M75). Numbers on the brackets indicate the relative values of the LUC/REN ratio of TT8/MYB75 with bHLH17 to that of TT8/MYB75. The LUC/REN ratio represents the *P_DFR_-LUC* activity relative to the internal control (*REN* driven by 35S promoter). Error bars represent SE (n = 3). Asterisks represent Student's t-test significance between pairs indicated with brackets (*, P<0.05; **, P<0.01). (C) Transient transcriptional activity assays show that activation of *DFR* promoter by TT8/MYB75 is repressed by bHLH3 and bHLH17. The *P_DFR_-LUC* reporter was cotransformed with the indicated constructs (10 µg for each construct). Error bars represent SE (n = 3). Asterisks represent Student's t-test significance (**, P<0.01). (D) The schematic diagrams show the constructs used in the transcriptional activity assays of (E). (E) Transient transcriptional activity assays show that activation of *TAT1* promoter by MYC2 is repressed by bHLH3 and bHLH17. The *P_TAT1_-LUC* reporter was cotransformed with the indicated constructs (10 µg for each construct). Error bars represent SE (n = 3). Asterisks represent Student's t-test significance (*, P<0.05; **, P<0.01).

We further generated a reporter construct *P_DFR_-LUC* in which the *LUC* reporter was driven by the promoter of *DFR* (Figure 9A), a direct target of the WD-repeat/TT8/MYB75 complex [46], [59], [60]. Consistent with previous data [61], [62], TT8 and MYB75 act as transcription activators to significantly induce expression of *P_DFR_-LUC* in the transient transcriptional activity assays (Figures 9A and 9B). We further found that bHLH17 repressed the TT8/MYB75-activated *P_DFR_-LUC* expression in a dosage-dependent manner (Figure 9B). Similarly, bHLH3 also repressed the TT8/MYB75-activated *P_DFR_-LUC* expression (Figure 9C).

Using similar approach, we generated a reporter construct *P_TAT1_-LUC* in which the *LUC* reporter was driven by the promoter of *TAT1* (Figure 9D), a direct target of MYC2 [63]. Consistent with previous data [63], MYC2 acts as transcription activator to significantly induce expression of *P_TAT1_-LUC* in the transient transcriptional activity assays (Figure 9E). Furthermore, we found that *bHLH3* and *bHLH17* were able to repress the MYC2-activated *TAT1* expression (Figures 9E). These results collectively suggest that the bHLH subgroup IIId factors antagonize transcription activators (such as MYC2, TT8 and MYB75) in JA pathway.

**Figure 10:**
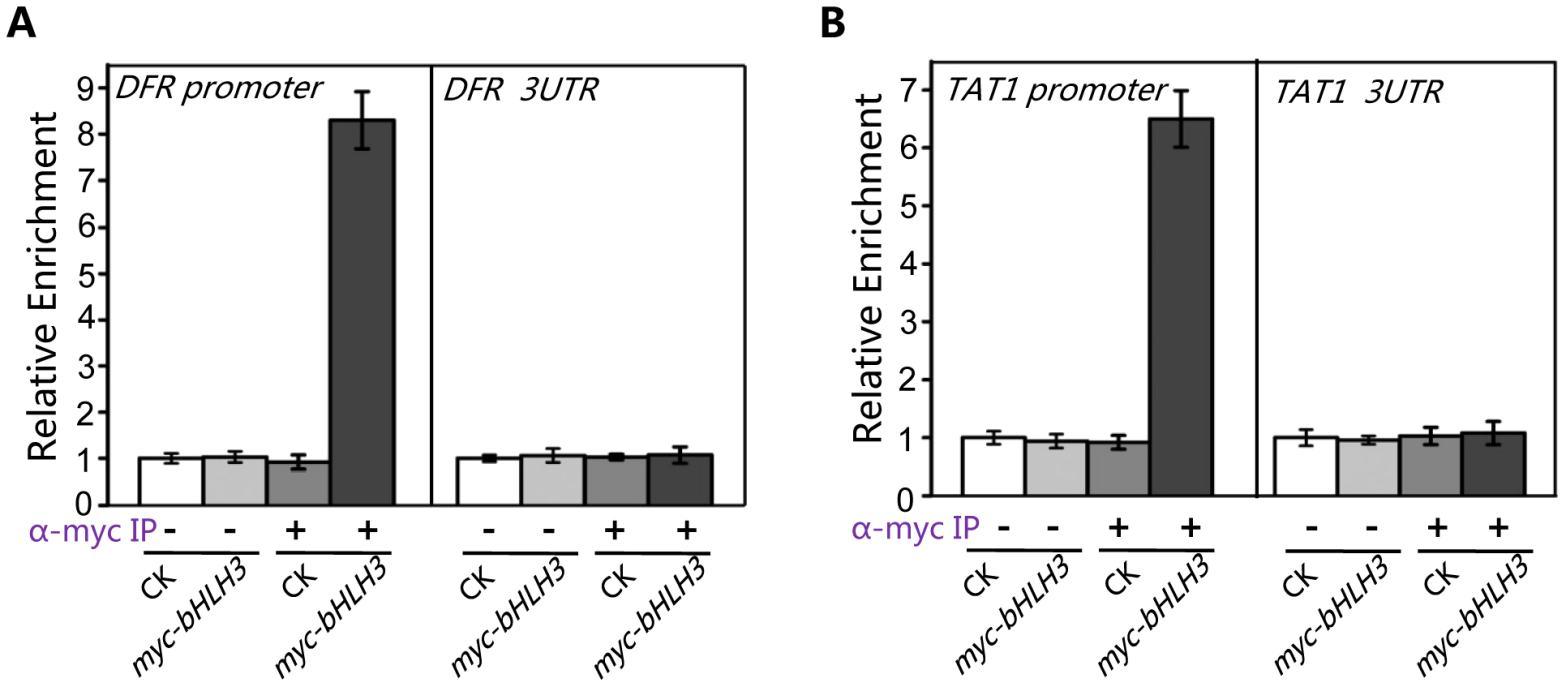
bHLH3 directly binds to promoter sequences of *TAT1* and *DFR* in ChIP-PCR assay. (A) ChIP-PCR analysis of the *in vivo* binding of myc-bHLH3 to the promoter of *DFR*. Chromatin from wild type plants (CK) and the transgenic plants constitutively expressing myc-bHLH3 (myc-bHLH3) was immunoprecipitated without (−) or with anti-myc antibody (+). Levels of the *DFR* promoter sequence (a 267-bp fragment containing two G-box motifs (CACGTG)) in the indicated chromatin were quantified by quantitative real-time PCR assay. PCR-amplification of a 77-bp 3′-UTR fragment of *DFR* was used as a negative control. The experiment was repeated three biological times with similar results. Error bars represent SE (n = 3). (B) ChIP-PCR analysis of the *in vivo* binding of myc-bHLH3 to the promoter of *TAT1*. Levels of the *TAT1* promoter sequence (a 111-bp fragment containing one G-box motif) in the indicated chromatin were quantified by quantitative real-time PCR assay. PCR-amplification of a 82-bp 3′-UTR fragment of *TAT1* was used as a negative control. The experiment was repeated three biological times with similar results. Error bars represent SE (n = 3).

The Y2H and BiFC assays detected no direct interactions between the transcription repressor (bHLH3, bHLH13, bHLH14 or bHLH17) and transcription activator (such as MYC2 or TT8/MYB75) (Figures S2, S3, S4), which excluded the possibility that the bHLH subgroup IIId factors antagonize the previously reported transcription activators via direct interactions. Previous studies showed the transcription activators TT8/MYB75 and MYC2 bind to and activate their respective target sequences, such as promoters of *DFR* and *TAT1* [61]–[63]. Here we used chromatin immunoprecipitation (ChIP)-PCR assays to investigate whether the bHLH subgroup IIId factors antagonize these transcription activators through binding to their target sequences. The ChIP-PCR assays showed that *DFR* promoter sequence, spanning two G-box motifs (CACGTG) at the position from −182 to −154, was highly enriched in the anti-myc-immunoprecipitated chromatin of the *myc-bHLH3* transgenic plant, but not in the controls (the anti-myc-pulled wild-type chromatin, the empty beads-pulled chromatin of wild-type or the *myc-bHLH3* transgenic plant) (Figure 10A), demonstrating a direct binding of myc-bHLH3 to the promoter sequence of *DFR*. Using the similar approach, we also detected the association of myc-bHLH3 with promoter of *TAT1* (Figure 10B).

Taken together, our results suggest that bHLH3, bHLH13, bHLH14 and bHLH17 factors function as transcriptional repressors to repress the JA responses. These transcriptional repressors antagonize the previously reported transcription activators through binding to their downstream target sequences.

## Discussion

The JAZ protéines [20], [29], [30], through formation of a core repression complex with TOPLESS and the NOVEL-INTERACTOR-OF-JAZ [57], are speculated to negatively regulate JA-mediated plant responses via interaction with and attenuation of their target transcription factors such as the bHLH subgroup IIIe transcription factors (MYC2, MYC3, MYC4) [29], [39]–[41], the R2R3-MYB transcription factors (MYB21, MYB24 and MYB57) [12], and the WD-repeat/bHLH (TT8, GL3 or EGL3)/MYB (MYB75 or GL1) complexes [14]. Here, we identified four bHLH subgroup IIId transcription factors (bHLH3, bHLH13, bHLH14 and bHLH17) as new targets of JAZ proteins (Figure 1). These transcription factors function as negative regulators to repress JA responses (Figures 4–7). Furthermore, our results showed that these negative regulators act as transcriptional repressors to antagonize the positive transcription factors through binding to their downstream target sequences (Figures 8–10). Coordinated regulation of JA responses by transcription repressors and transcription activators may benefit plants for adaptation to their frequently changing nature habitat.

Genetic and physiological analysis on the single, double, triple and quandruple mutants (*bhlh3*, *bhlh13*, *bhlh14*, *bhlh17*, *bhlh3 bhlh17*, *bhlh3 bhlh13 bhlh17*, *bhlh3 bhlh13 bhlh14*, *bhlh3 bhlh14 bhlh17*, *bhlh13 bhlh14 bhlh17*, *bhlh3 bhlh13 bhlh14 bhlh17*) showed that these bHLH subgroup IIId transcripiton factors function redundantly to repress JA responses (Figures 3–6 and S1). The quandruple mutant *bhlh3 bhlh13 bhlh14 bhlh17* exhibited significant increase in JA responses, while the single mutant *bhlh3*, *bhlh13*, *bhlh14* or *bhlh17* displayed no obvious alterations in the tested JA responses (Figures 3–6 and S1), though we cannot exclude possibility that some JA responses may be mildly altered in single mutants. As shown in Figure 2 C and D, *bhlh3* showed a mild increase in JA-inducible anthocyanin accumulation. Recent study also showed that the single mutant *bhlh17*/*jam1* (another T-DNA insertion mutant of *bHLH17/JAM1*) displayed no obvious alteration in JA-inhibitory root growth, but exhibited the enhanced sensitivity in JA-inducible anthocyanin accumulation and defense against insect [64]. The functional redundancy among these bHLH3, bHLH13, bHLH14 and bHLH17 factors may result from their high similarity at amino acid level. It would be interesting to investigate whether and how these transcription factors exhibit homo- and heterodimerization to exert their redundant functions.

In addition to their redundant function, it is not clear whether the bHLH3, bHLH13, bHLH14 and bHLH17 factors have distinct roles in regulation of JA responses. The bHLH13 and bHLH17 exhibited similar interaction patterns with 10 JAZ proteins, and with the Jas domain of JAZ8/JAZ11 (Figure 1). However, for bHLH3 and bHLH14, they interacted with 7 JAZ proteins, and the full length of JAZ8/JAZ11 was required for interactions with bHLH3 and bHLH14 (Figure 1). Furthermore, the *COI1*-dependent and JA-induced gene expression was observed for *bHLH13* and *bHLH17*, but not for *bHLH3* and *bHLH14* (Figure 2D). Interestingly, both bHLH3 and bHLH17 were nucleus-localized (Figure 2C), while bHLH13 and bHLH14 were localized in both nucleus and cytoplasm (Figure 2C). It remains to be elucidated whether these distinguished features would lead to distinct roles for these four transcription factors in JA pathway. Current studies so far have showed that bHLH17/AtAIB was positively involved in ABA signaling [43]. It is not clear whether the bHLH subgroup IIId factors play positive or negative roles in other signal pathways.

Previous studies showed that MYC2, and the WD-repeat/bHLH (TT8, GL3 or EGL3)/MYB (MYB75 or GL1) complexes act as transcription activators which bind to and activate promoter sequences of their respective target genes, such as *TAT1* [63], and *DFR* [46], [60], [61]. We showed that the bHLH subgroup IIId factors act as transcription repressors (Figure 8), which bind to the promoter sequences of *TAT1 and DFR* (Figure 9 and 10), to antagonize the transcription function of MYC2 and MYB75/TT8 (Figure 9). It is interesting to investigate whether the bHLH subgroup IIId factors antagonize the activation function of MYC2 and MYB75/TT8 through competitive or concurrent binding to these target promoter sequences. Fernandez-Calvo et al. predicted that the activation domain is localized at the N-terminus of MYC2 [40]. It remains to experimentally investigate which domain in MYC2 (or in the bHLH subgroup IIId factors) is responsible for activation (or repression) of their target sequences.

The GAL4DB-based protoplast transient expression system is a well-established method for determination of transcription activators and repressors [57], [58], [65]–[68]. MYC2 acts as a transcription activator in this transient expression system (Figure 8A and 8B), which is consistent with previous observations [39], [57], [63], [69]. Consistent with its activation function, the *MYC2* is a key gene that positively regulates a vast array of JA-responsive genes and diverse aspects of JA responses, including root growth [22], [70], anthocyanin accumulation [71], sesquiterpene synthase [72], nicotine biosynthesis [73], wound response [74], oxidative stress tolerance [75], and plant defense against both bacterial pathogen *Pst* DC3000 [40] and insects (*Spodoptera littoralis* and *Helicoverpa armigera*) [40], [75]. Interestingly, the *MYC2* gene was shown to negatively regulate defense against necrotrophic fungi (*B. cinerea* and *Plectosphaerella cucumerina*) and expression of the related genes, such as *PDF1.2* [22], [75], [76]. We showed that the bHLH subgroup IIId factors act as transcription repressors (Figures 8), which antagonize the activation function of MYC2 and TT8/MYB75 (Figure 9), to negatively regulate all the tested JA responses, including root growth, flowering, anthocyanin accumulation, and plant defense responses against bacterial pathogen *Pst* DC3000, necrotrophic pathogen *B. cinerea*, and insect *S. exigua* (Figures 4–7). Generation and characterization of the penta mutant *myc2 bhlh3 bhlh13 bhlh14 bhlh17* would clarify whether mutations in the bHLH subgroup IIId factors are able to rescue the *myc2-*associated reduction of JA responses (such as root growth, anthocyanin accumulation, wound response, and defense against bacterial pathogen and insects), and to additively or synergistically affect the *myc2-*associated increase of defense response against necrotrophic pathogen *B. cinerea*.

**Figure 11:**
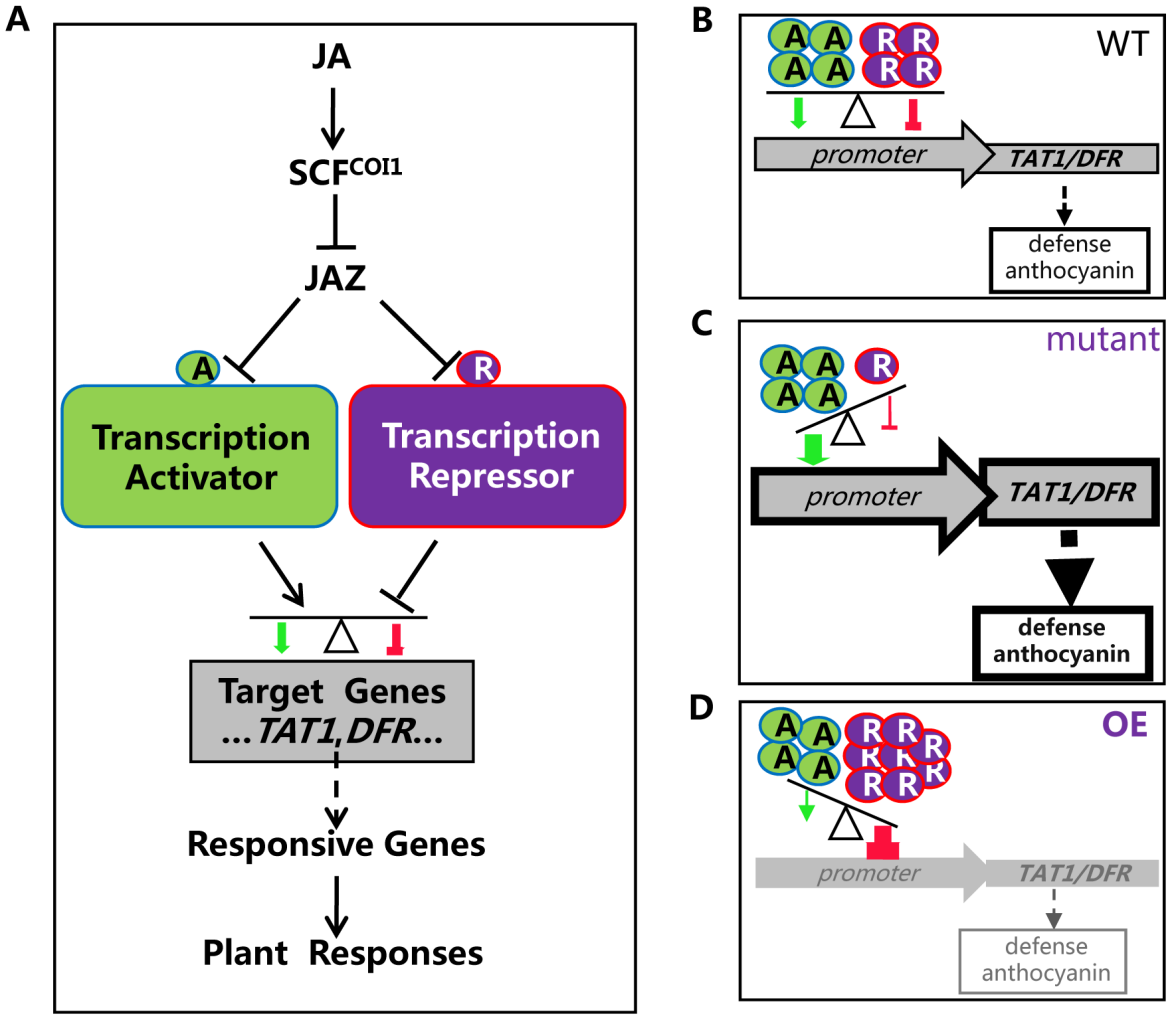
A simplified model for coordinated regulation of JA responses by JAZ-targeted transcription activators and transcription repressors. (A) JAZ proteins interact with transcription activators (such as MYC2, TT8 and MYB75) and transcription repressors (including bHLH3, bHLH13, bHLH14 and bHLH17) to attenuate their transcriptional function. Upon perception of JA, SCF^COI1^ recruits JAZs for ubiquitination and degradation via 26S proteasome. The JAZ-targeted transcription activators and repressors are then released to antagonistically and coordinately regulate their target genes (such as *TAT1* and *DFR*), which may further regulate JA responsive genes essential for various JA responses. (B) In wild type plant, both transcription activators and repressors, in response to JA signal, are released to regulate expression of their mutual target genes (such as *TAT1* and *DFR*). The balance between repression and activation would lead to an appropriate output of JA-responsive genes, resulting in an appropriate level of JA responses such as plant defense and anthocyanin accumulation. A, transcription activator; R, transcription repressor. (C) In the *bhlh3 bhlh13 bhlh14 bhlh1*7 quadruple mutant, the transcription repressors were severely abolished. The balance between repression and activation would lead to enhanced output of JA-responsive genes, resulting in increased JA responses. (D) In the plants overexpressing the bHLH subgroup IIId factors (OE), high level of the transcription repressors would exhibit strongest repression. The balance between repression and activation would lead to decreased output of JA-responsive genes, resulting in reduced JA responses.

It is speculated that, in response to JA signal, JAZ proteins are recruited by SCF^COI1^ for ubiquitination and subsequent degradation. As a result, the JAZ-targeted transcription activators and repressors (bHLH3, bHLH13, bHLH14 and bHLH17) are released to antagonistically and coordinately regulate their target genes (such as *TAT1* and *DFR*), which may further modulate expression of JA responsive genes essential for various JA responses (Figure 11). Plants live in fixed places and have to evolve sophisticated systems for adaptation to their frequently changing environment. The antagonistic and coordinated regulation of JA responses by transcription repressors and transcription activators may provide an important strategy for plant survival in their complicated nature habitat. It is possible that plants evolve this type of repressor-mediated negative regulation system to provide a fine feedback regulatory mechanism for avoiding exhausted and harmful excess JA responses.

## Materials and Methods

### Plant Materials and Growth Conditions

The *Arabidopsis thaliana* mutant *coi1-1* [77] was described as previously. *bhlh3* (CS428877/GK-301G05), *bhlh13* (CS466724/GK-696A04), *bhlh14* (CS27164/GT1193.Ds3.05.24.99.b.288) and *bhlh17* (CS874647/SAIL_536_F09) were obtained from the ABRC or NASC. The double, triple and quadruple mutants of the bHLH subgroup IIId factors were generated through genetic crossing among *bhlh3*, *bhlh13*, *bhlh14* and *bhlh17*.

*Arabidopsis thaliana* seeds were sterilized with 20% bleach, plated on Murashige and Skoog medium (MS; Sigma-Aldrich), chilled at 4°C for 3 days, and transferred to a growth room under a 16-h (22–24°C)/8-h (16–19°C) light/dark photoperiod. *Nicotiana benthamiana* was grown in a growth room under a 16-h (28°C)/8-h (22°C) light/dark condition.

### Y2H Screening and Y2H Assays

The yeast two-hybrid screening method was described as previously [12], [14].

For Y2H assay, all the CDS of *JAZ*s, *bHLH3*, *bHLH13*, *bHLH14*, *bHLH17*, *MYC2*, *MYC3*, *MYC4*, *TT8*, *GL3*, *EGL3*, *MYB75*, *GL1* and *TTG1*, and their domain derivatives were cloned into pLexA or pB42AD vectors. Primers used for the vector construction are presented in Table S1. The method details for yeast transformation and interaction detection using EGY48 were described as previously [12], [14]. Y2H images were taken 3 days after incubation at 30°C. Experiments were repeated three biological times.

### BiFC Assays

For BiFC assays, full-length coding sequences of *Arabidopsis JAZ1*, *JAZ10*, *bHLH3*, *bHLH13*, *bHLH14*, *bHLH17*, *MYC2*, *MYC3*, *MYC4*, *TT8*, *GL3*, *EGL3*, *MYB75* and *GL1* were cloned into the binary nYFP or cYFP vector through enzyme digestion sites (KpnI/SalI) or Gateway reaction with pDONR207 vector system (Invitrogen) [12], [14]. Primer pairs for generation of constructs are listed in Table S1. *Agrobacterium* strains with indicated nYFP or cYFP vector were incubated, harvested, and resuspended in infiltration buffer (0.2 mM acetosyringone, 10 mM MgCl_2_, and 10 mM MES). Equal concentrations and volumes of *Agrobacterium* strains were mixed and coinfiltrated into *N. benthamiana* leaves by a needleless syringe. After infiltration, plants were placed at 24°C for 50 h before observation. The experiments were repeated three biological times.

### Quantitative Real-time PCR

In Figure 2B, root, stem, rosette leaf, stem leaf and flowers were harvested for RNA extraction and subsequent reverse transcription. In Figures 2D and 7E, *Arabidopsis* seedlings were grown on MS medium for 11 days and then were treated with or without 100 µM MeJA for indicated time. In Figures 4B, 4E, 5E and 7D, *Arabidopsis* seedlings were grown on MS medium supplied with or without indicated concentration of MeJA for eleven days. These materials were harvested for RNA extraction and subsequent reverse transcription.

Real-time PCR analyses were performed with the RealMasterMix (SYBR Green I) (Takara) using the ABI7500 real-time PCR system as described previously [14]. *ACTIN8* was used as the internal control. The primers used for real-time PCR analysis are presented in Table S2. All the experiments were repeated three biological times with similar results.

### Protoplast Transfection Assays

For transient expression assay in *Arabidopsis* protoplast using the GUS reporter, the CDS of *bHLH3*, *bHLH13*, *bHLH14*, *bHLH17* and *MYC2* were fused with the GAL4DB under control of 35S promoter. The coding sequence of *JAZ1* was cloned into the pGreenII 62-SK vector under control of 35S promoter [78]. Primers used for plasmid construction were shown in Table S1. Four copies of upstream GAL4 DNA binding sites (GAL4(4x)-D1-3(4x)) were used to drive the GUS gene generating the GUS reporter construct [58], [68]. The internal control contains a *firefly luciferase* gene (*LUC*) under control of 35S promoter. *Arabidopsis* mesophyll protoplasts preparation and subsequent transfection were performed as described previously [79]. Relative GUS activity was normalized against the LUC activity. In the JAZ1 dosage effect test of Figure 8B, 0.2, 1, 5 or 10 µg JAZ1 plasmid was respectively used, while for other reporter and effectors, 10 µg plasmid was used.

For transient transcriptional activity assay using the LUC reporter, the CDS sequences of *MYC2*, *TT8*, *MYB75*, *bHLH3* and *bHLH17* were cloned into pGreenII 62-SK vectors under control of 35S promoter respectively. The ∼519 bp and ∼980 bp promoter sequences of *DFR* and *TAT1* were amplified from genomic DNA and cloned into pGreenII 0800-LUC respectively [78]. All primers used for making these constructs are listed in Table S1. After protoplasts preparation and subsequent transfection, firefly luciferase (LUC) and renillia luciferase (REN) activities were measured using the Dual-Luciferase Reporter Assay System (Promega) following the manufacturer's instructions. Relative firefly luciferase (LUC) activity was calculated by normalizing against the renillia luciferase activity. In the experiment of bHLH17 dosage effect in Figure 9B, 0.2, 1, 5 or 10 µg bHLH17 plasmid was respectively used, while for other reporter and effectors, 10 µg plasmid was individually used. All the experiments were repeated three biological times with similar results.

### ChIP Assays

The ChIP experiment was performed as described previously [80] using leaves of the 4-week-old *myc*-*bHLH3* transgenic plants and the wild-type (Col-0) plants treated with 100 µM MeJA for 40 minutes. Immunoprecipitation was performed using Mouse anti-MYC antibody and protein G agarose beads. Enrichment of promoter DNA was confirmed by qRT-PCR using *ACTIN2* as normazlization control. Primers for 3′UTR region were used as negative control. Primers for the ChIP assays are listed in Table S2. The experiments were repeated for three biological repeats with similar results.

### Generation of Transgenic Plants

To generate *Arabidopsis* transgenic plants overexpressing *bHLH13* and *bHLH17*, the full-length coding sequences of *bHLH13* and *bHLH17* were amplified and cloned into the modified pCAMBIA1300 vector under control of 35S promoter through the SalI and SpeI sites. To generate *Arabidopsis* myc-bHLH3 transgenic plants, coding sequence of *bHLH3* was cloned into pROK2-myc vectors for fusion with six myc tags. These constructs were introduced into *Arabidopsis* plants using the *Agrobacterium*-mediated floral dip method. Two representative transgenic lines for *bHLH13* and *bHLH17* respectively were displayed in the article.

### GUS Staining

∼2520 bp, ∼2500 bp, ∼1050 bp and ∼2570 bp promoter regions of *bHLH3*, *bHLH13*, *bHLH14* and *bHLH17* were respectively amplified and cloned into pCAMBIA1391Z vector to drive the *GUS* genes for generation of *P_bHLH3_-GUS*, *P_bHLH13_-GUS*, *P_bHLH14_-GUS* and *P_bHLH17_-GUS*. These constructs were transformed into *Agrobacterium* strains GV3101, and transferred into *Arabidopsis* by floral dip methods. Seedlings, inflorescences and roots from the transgenic plants harboring *P_bHLH3_-GUS*, *P_bHLH13_-GUS*, *P_bHLH14_-GUS* or *P_bHLH17_-GUS* were used for histochemical staining of GUS. Histochemical staining for GUS activity assay was performed as described previously [18].

### Subcellular Localization

Coding sequences of *bHLH3*, *bHLH13*, *bHLH14* and *bHLH17* were respectively cloned into pEGAD vector for fusion with GFP under control of 35S promoter to generate the *GFP-bHLH3*, *GFP-bHLH13*, *GFP-bHLH14* and *GFP-bHLH17* constructs. The *Agrobacterium* containing the indicated constructs were resuspended in infiltration buffer (0.2 mM acetosyringone, 10 mM MgCl_2_, and 10 mM MES), and infiltrated into *N. benthamiana* leaves by a needleless syringe. After infiltration, plants were placed at 24°C for 50 h before GFP observation.

### Anthocyanin Measurement

For anthocyanin measurement, 11-d-old *Arabidopsis* seedlings grown on MS medium with 0, 5, or 15 µM MeJA were measured as described previously [14] The anthocyanin content is presented as (A535–A650)/g fresh weight. The experiment was repeated three biological times.

### Root Length Measurement

Seeds were grown on MS medium with 0, 5, 20 or 50 µM MeJA, chilled at 4°C for 3 days, and transferred to the growth room. Root lengths of fifteen 11-d-old seedlings for each genotype and treatment were measured and presented. The experiment was repeated three biological times.

### Flowering Time

Flowering time of plants, grown in soil under long day condition with 16-h(22–25°C)/8-h(18–21°C) light/dark photoperiod, was recorded as the number of days from germination to the first appearance of buds in the rosette center. The experiment was repeated three biological times.

### Infection with Pathogens

For Figures 5A and 5C, thirty-day-old plants were sprayed with *Botrytis cinerea* (10^6^ spores/mL) solved in 0.025% tween or with 0.025% tween as control, placed in dark at the appropriate temperature (22°C) and high humidity (100%) for 36 hours, and transferred to a growth room under the growth conditions of a 16-h-light (21 to 23°C)/8-h-dark (16 to 19°C) photoperiod with ∼40% humidity. Infection symptoms were recorded at 7-day after infection. Infection ratings from 0 to 3 were assigned to the inoculated plants (0, no visible symptoms; 1, weak symptoms; 2, severe symptoms; 3, dead plants). For Figures 5B and 5D, four-week-old plants were sprayed with Botrytis cinerea (10^7^ spores/mL) solved in 0.025% tween or with 0.025% tween as control, placed in dark at the appropriate temperature (22°C) and high humidity (100%) for 36 hours, transferred to a growth incubator under the growth conditions of a 16-h-light (21 to 23°C)/8-h-dark (16 to 19°C) photoperiod with high humidity (>90%). Plant survival ratio were recorded at 9-day after infection. At least thirty plants from each genotype were used in each experiment. The experiment was repeated three biological times.

Thirty-day-old plants were sprayed with *Pseudomonas syringae* pv *tomato* (*Pst*) DC3000 suspension containing 10^8^ (colony-forming units)/mL bacteria (OD600 = 0.2) with 0.02% Silwet L-77 or 0.02% Silwet L-77 as control. Infection symptoms were recorded at 3-day after infection. At least thirty plants from each genotype were used in each experiment. The bacterial population counts in the plant was determined as previously described [81]. The experiment was repeated three biological times.

### Insect Defense Assay with *Spodoptera exigua*

More than fifty mature rosette leaves with similar size from 4-week-old plants for each genotype were placed in one plastic Petri dishes (90 mm) containing wet filter paper. The 10 third-instar *S. exigua* larvae (∼8 mg each) were weighted, and reared on leaves in one Petri dish, for each genotype using five independent replicates. Two days after feeding, the weight of the 10 larvae were measured. The increase of average larval weight was recorded.

In the *two-choice test*, five rosette leaves from 4-week-old plants for each genotype were placed intervally in a circle in plastic Petri dishes (90 mm) containing wet filter paper. 40 newly hatched larvae were placed in the center of the Petri dishes for equal distance to the leaves. One day after incubation in the growth room, the numbers of larvae on leaves for each genotype were recorded. Four independent replicates were performed.

In the *three-choice test*, three rosette leaves from 4-week-old plants for each genotype were placed intervally in a circle in plastic Petri dishes (90 mm) containing wet filter paper. 40 newly hatched larvae were placed in the center of the Petri dishes for equal distance to the leaves. One day after incubation in the growth room, the numbers of larvae on leaves for each genotype were recorded. Six independent replicates were performed.

### Accession Numbers

The Arabidopsis Genome Initiative numbers for genes mentioned in this article are as follows: JAZ1 (AT1G19180), JAZ2 (AT1G74950), JAZ3 (AT3G17860), JAZ4 (AT1G48500), JAZ5 (AT1G17380), JAZ6 (AT1G72450), JAZ7 (AT2G34600), JAZ8 (AT1G30135), JAZ9 (AT1G70700), JAZ10 (AT5G13220), JAZ11 (AT3G43440), JAZ12 (AT5G20900), bHLH3 (AT4G16430), bHLH13 (AT1G01260), bHLH14 (AT4G00870), bHLH17 (AT2G46510), MYC2 (AT1G32640), MYC3 (AT5G46760), MYC4 (AT4G17880), TT8 (AT4G09820), GL3 (AT5G41315), EGL3 (AT1G63650), MYB75 (AT1G56650), GL1 (AT3G27920), TTG1 (AT5G24520), DFR (AT5G42800), LDOX (AT4G22880), UF3GT (AT5G54060), VSP1 (AT5G24780), THI2.1 (AT1G72260), PDF1.2 (AT5G44420), LOX2 (AT3G45140), TAT1 (AT4G23600), ERF1 (AT3G23240) and ACTIN8 (AT1G49240).

## Supporting Information

Figure S1 *bHLH3*, *bHLH13*, *bHLH14* and *bHLH17* Function Redundantly to Repress JA-regulated Anthocyanin Accumulation. Anthocyanin contents of the 11-day-old seedlings in WT, the single *bHLH* mutants (*bhlh3*, *bhlh13*, *bhlh14* and *bhlh17*), the triple mutants (*bhlh3,13,14*, *bhlh3,13,17*, *bhlh3,14,17* and *bhlh13,14,17*), the quadruple mutant *bhlh3,13,14,17* (*Q4*) and *coi1-1* grown on MS medium (MS) or MS medium containing 5 or 15 µM MeJA. FW, fresh weight. Error bars represent SE (n = 3). Asterisks denote Student's t-test significance compared with WT plants: **, *P*<0.01.
(TIF)

Figure S2 bHLH3, bHLH13 and bHLH17 Cannot Interact with MYC2, MYC3, MYC4, GL3, EGL3, TT8, MYB75, GL1 and TTG1 in Y2H Assays.
(TIF)

Figure S3 bHLH3, bHLH13, bHLH14 and bHLH17 Cannot Interact with MYC2, MYC3 and MYC4 in *N. benthamiana* in BiFC Assays.
(TIF)

Figure S4 bHLH3, bHLH13, bHLH14 and bHLH17 Cannot Interact with GL3, EGL3, TT8, MYB75 and GL1 in *N. benthamiana* in BiFC Assay
(TIF)

Table S1 Primers Used for Vector Construction.
(DOC)

Table S2 Primers Used for Quantitative Real-time PCR Analysis and ChIP PCR.
(DOC)
