# Supplementary figures and images for: The bHLH Subgroup IIId Factors Negatively Regulate Jasmonate-Mediated Plant Defense and Development
Source: PLoS Genet. 2013 Jul 25;9(7):e1003653. doi: 10.1371/journal.pgen.1003653 (PMC3723532; doi:10.1371/journal.pgen.1003653)

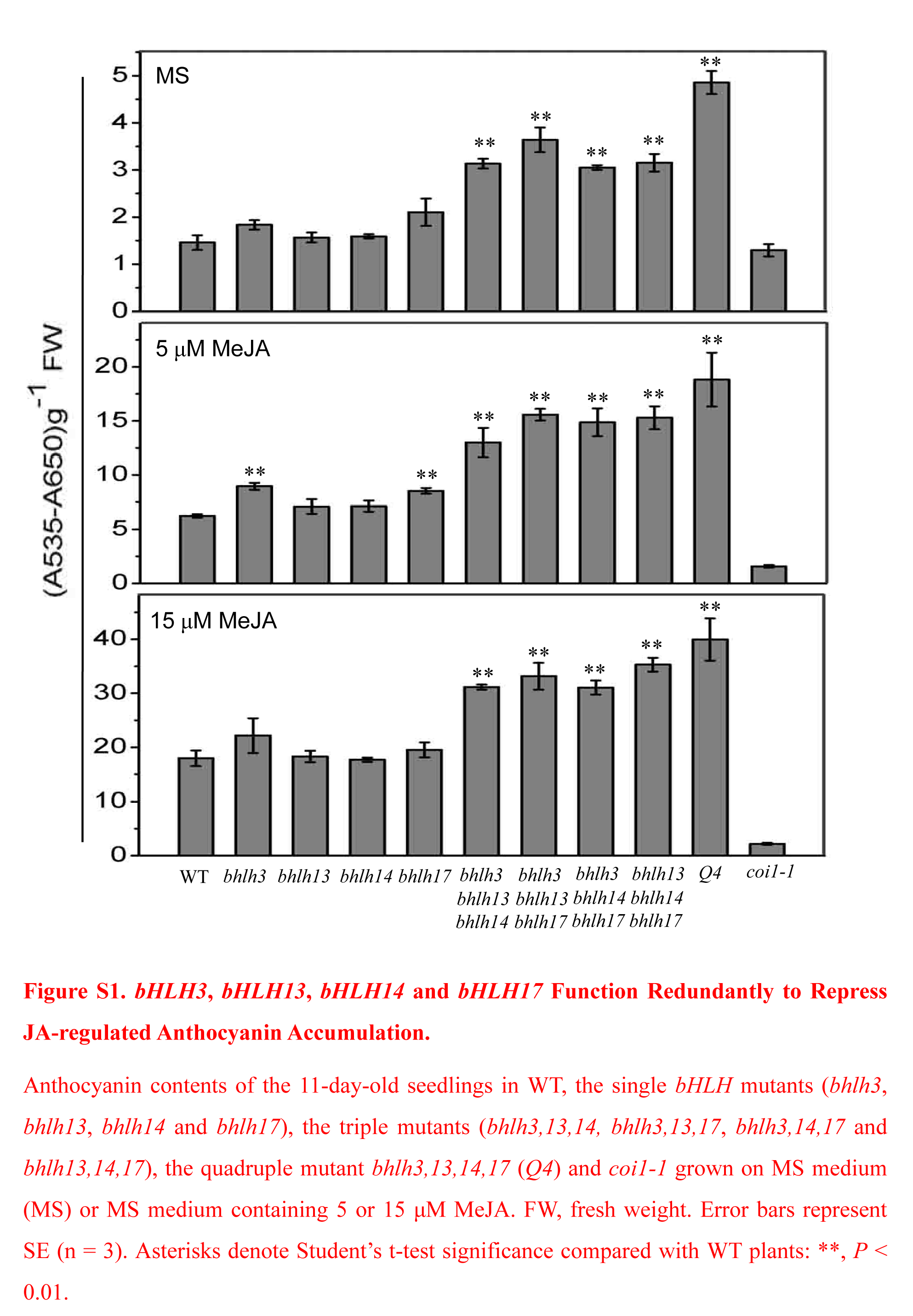

Supplement: Figure S1 — bHLH3, bHLH13, bHLH14 and bHLH17 Function Redundantly to Repress JA-regulated Anthocyanin Accumulation. Anthocyanin contents of the 11-day-old seedlings in WT, the single bHLH mutants (bhlh3, bhlh13, bhlh14 and bhlh17), the triple mutants (bhlh3,13,14, bhlh3,13,17, bhlh3,14,17 and bhlh13,14,17), the quadruple mutant bhlh3,13,14,17 (Q4) and coi1-1 grown on MS medium (MS) or MS medium containing 5 or 15 µM MeJA. FW, fresh weight. Error bars represent SE (n = 3). Asterisks denote Student's t-test significance compared with WT plants: **, P<0.01. (TIF) [file pgen.1003653.s001.tif]

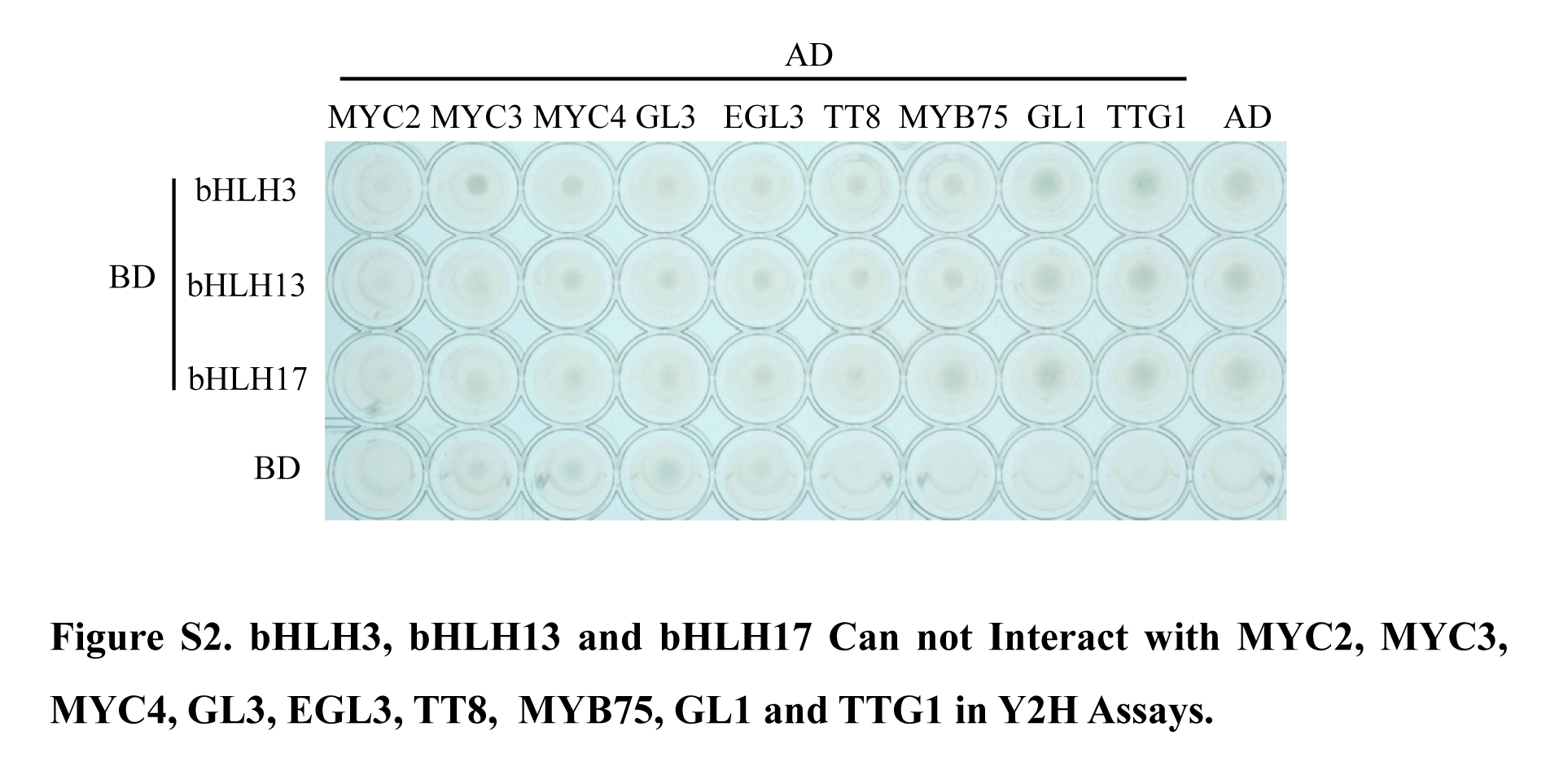

Supplement: Figure S2 — bHLH3, bHLH13 and bHLH17 Cannot Interact with MYC2, MYC3, MYC4, GL3, EGL3, TT8, MYB75, GL1 and TTG1 in Y2H Assays. (TIF) [file pgen.1003653.s002.tif]

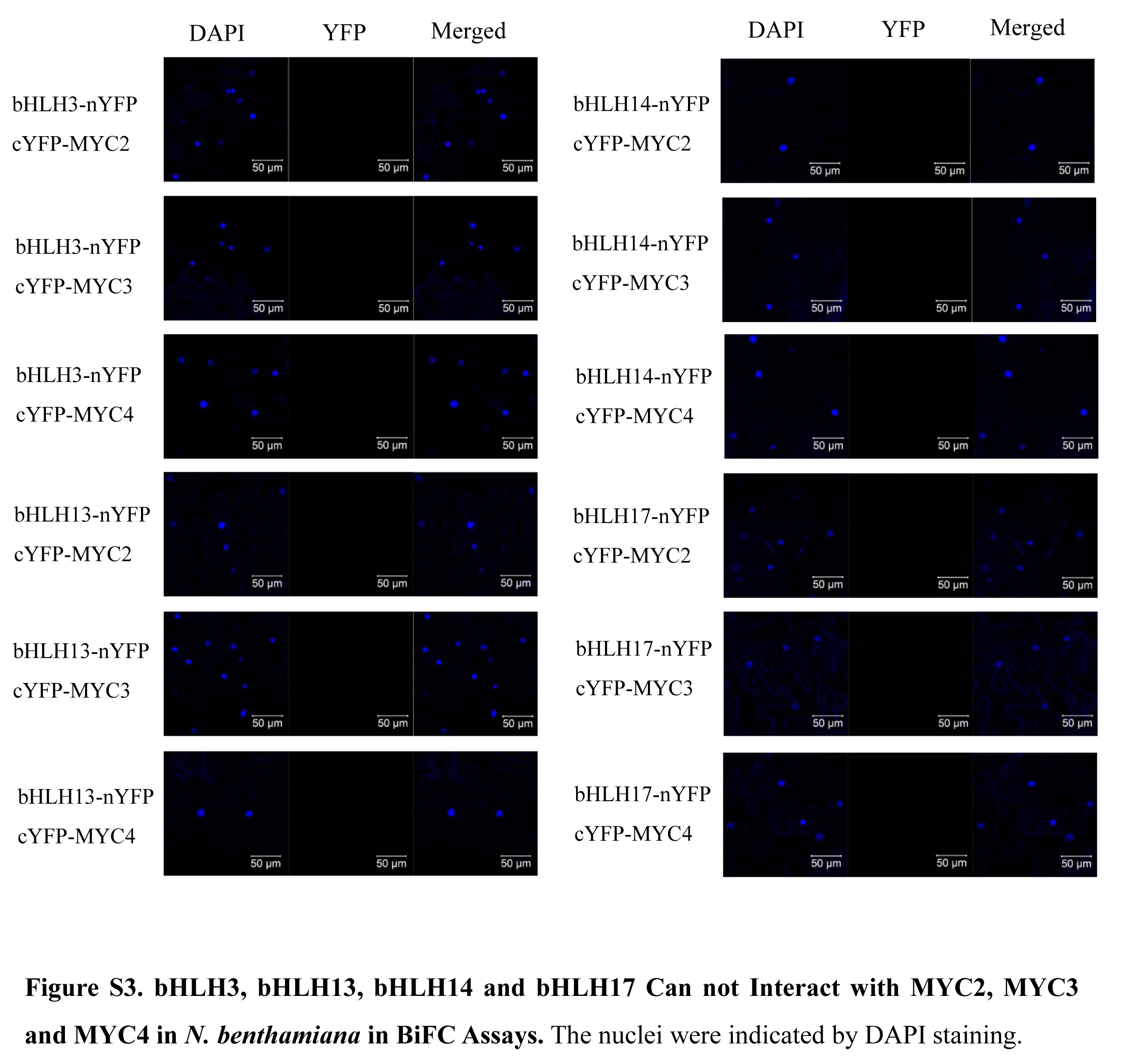

Supplement: Figure S3 — bHLH3, bHLH13, bHLH14 and bHLH17 Cannot Interact with MYC2, MYC3 and MYC4 in N. benthamiana in BiFC Assays. (TIF) [file pgen.1003653.s003.tif]

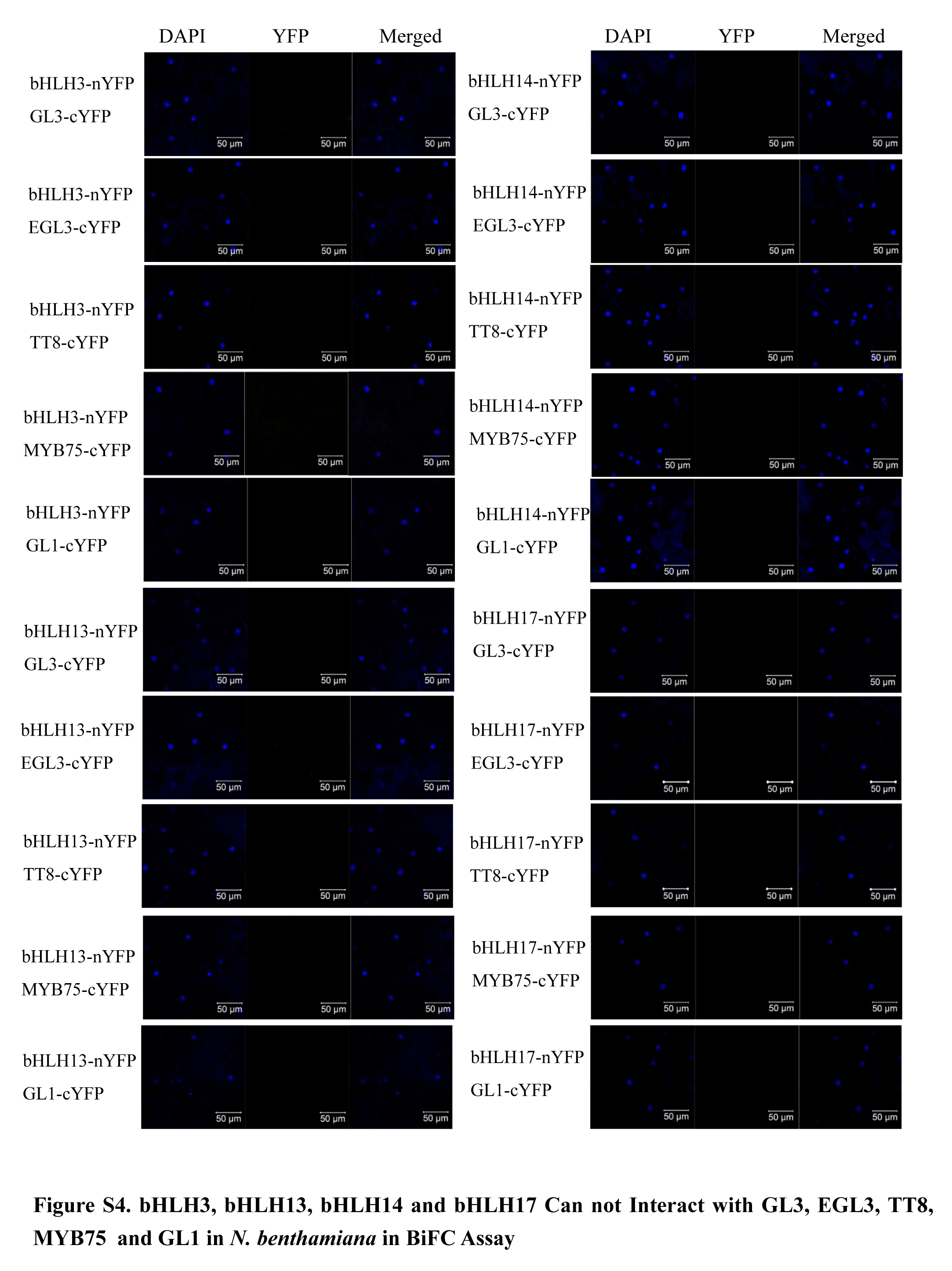

Supplement: Figure S4 — bHLH3, bHLH13, bHLH14 and bHLH17 Cannot Interact with GL3, EGL3, TT8, MYB75 and GL1 in N. benthamiana in BiFC Assay (TIF) [file pgen.1003653.s004.tif]
