# Supplementary material for: The bHLH Subgroup IIId Factors Negatively Regulate Jasmonate-Mediated Plant Defense and Development
Source: PLoS Genet. 2013 Jul 25;9(7):e1003653. doi: 10.1371/journal.pgen.1003653 (PMC3723532; doi:10.1371/journal.pgen.1003653)
Supplement: Table S1 — Primers Used for Vector Construction. (DOC) [file pgen.1003653.s005.doc]

**Table S1. Primers Used for Vector Construction.**

| JAZ1 pLexA | Forward-EcoRI | cccgaattcatgtcgagttctatggaatg |
| --- | --- | --- |
| JAZ1 pLexA | Reverse-SalI | aaaagtcgactcatatttcagctgctaaacc |
| JAZ2 pLexA | Forward-EcoRI | cccgaattcatgtcgagtttttctgccgagtg |
| JAZ2 pLexA | Reverse-XhoI | accgctcgagttaccgtgaactgagccaagctg |
| JAZ3 pLexA | Forward-EcoRI | ggggaattcatggagagagattttctcgggttg |
| JAZ3 pLexA | Reverse-XhoI | cccgctcgagttaggttgcagagctgagagaag |
| JAZ4 pLexA | Forward-EcoRI | ggggaattc atggagagagattttctcgggctg |
| JAZ4 pLexA | Reverse-XhoI | cccgctcgagttagtgcagatgatgagctggag |
| JAZ5 pLexA | Forward-EcoRI | cccgaattcatgtcgtcgagcaatgaaaatgc |
| JAZ5 pLexA | Reverse-XhoI | cccgctcgagctatagccttagatcgagatc |
| JAZ6 pLexA | Forward-EcoRI | ggggaattcatgtcaacgggacaagcgccggag |
| JAZ6 pLexA | Reverse- XhoI | cccgctcgagctaaagcttgagttcaaggtt |
| JAZ7 pLexA | Forward-EcoRI | cccgaattcatgatcatcatcatcaaaaactg |
| JAZ7 pLexA | Reverse-XhoI | accgctcgagctatcggtaacggtggtaagg |
| JAZ8 pLexA | Forward-NcoI | ccccccatggatgaagctacagcaaaattgtg |
| JAZ8 pLexA | Reverse-XhoI | gggctcgagttatcgtcgtgaatggtacggtg |
| JAZ9 pLexA | Forward-EcoRI | ggggaattc atggaaagagattttctgggtttg |
| JAZ9 pLexA | Reverse-XhoI | ccgctcgagttatgtaggagaagtagaagag |
| JAZ10 pLexA | Forward-EcoRI | ggggaattcatgtcgaaagctaccatagaactcg |
| JAZ10 pLexA | Reverse-SalI | acgcgtcgac ttaggccgatgtcggatagtaag |
| JAZ11 pLexA | Forward-EcoRI | ggggaattcatggctgaggtaaacggagatttc |
| JAZ11 pLexA | Reverse-SalI | aaaagtcgactcatgtcacaatggggctgg |
| JAZ12 pLexA | Forward-BamHI | cccggatccatgactaaggtgaaagatgagcc |
| JAZ12 pLexA | Reverse-SalI | acgcgtcgacctaagcagttggaaattcctcc |
| JAZ8NT pLexA | Forward-NcoI | ccccccatggatgaagctacagcaaaattgtg |
| JAZ8NT pLexA | Reverse-XhoI | ccgctcgagttatggatttggaagctgattatg |
| JAZ8CT pLexA | Forward-NcoI | ccccccatgggaaatgaaaaccaagtcatcc |
| JAZ8CT pLexA | Reverse-XhoI | gggctcgagttatcgtcgtgaatggtacggtg |
| JAZ8Jas pLexA | Forward-BamHI | cgcggatccagcttccaaatccaaaagcatc |
| JAZ8Jas pLexA | Reverse-XhoI | gggctcgagttatcgtcgtgaatggtacggtg |
| JAZ11NT pLexA | Forward-EcoRI | ggggaattcatggctgaggtaaacggagatttc |
| JAZ11NT pLexA | Reverse-SalI | aaaagtcgactcatgctgtcgatacgcaagctac |
| JAZ11CT pLexA | Forward-EcoRI | ggggaattcaaagccactgagacaattaatt |
| JAZ11CT pLexA | Reverse-SalI | aaaagtcgactcatgtcacaatggggctgg |
| bHLH3 pB42AD | Forward-MfeI | ccccaattgatgggtcaaaagttttgggag |
| bHLH3 pB42AD | Reverse-XhoI | cccgctcgagttactgtgatagagaggcaag |
| bHLH13 pB42AD | Forward-EcoRI | ggggaattcatgaatattggtcgcctagtgtgg |
| bHLH13 pB42AD | Reverse-SalI | acgcgtcgacctatctacctgatgatgttcttgac |
| bHLH14 pB42AD | Forward-EcoRI | ggggaattcatgtataatctcactttctctcc |
| bHLH14 pB42AD | Reverse-XhoI | cccgctcgagttataggcttagggttcggacaag |
| bHLH17 pB42AD | Forward-EcoRI | ggggaattcatgaatatgagtgatttagg |
| bHLH17 pB42AD | Reverse-XhoI | cccgctcgagttatatatcaccagagacctg |
| bHLH13NT pB42AD | Forward -EcorI | ggggaattcatgaatattggtcgcctagtgtgg |
| bHLH13NT pB42AD | Reverse-SalI | acgcgtcgacctatgaactcgcagcagagaaatc |
| bHLH13CT pB42AD | Forward -EcorI | ggggaattcctgcttcctcctgctcaaatg |
| bHLH13CT pB42AD | Reverse-XhoI | cccgctcgagctatctacctgatgatgttcttgac |
| bHLH3 pLexA | Forward-MfeI | ccccaattgatgggtcaaaagttttgggag |
| bHLH3 pLexA | Reverse-XhoI | cccgctcgagttactgtgatagagaggcaag |
| bHLH13 pLexA | Forward-EcoRI | ggggaattcatgaatattggtcgcctagtgtgg |
| bHLH13 pLexA | Reverse-SalI | acgcgtcgacctatctacctgatgatgttcttgac |
| bHLH17 pLexA | Forward-EcoRI | ggggaattcatgaatatgagtgatttagg |
| bHLH17 pLexA | Reverse-XhoI | cccgctcgagttatatatcaccagagacctg |
| MYC2 pB42AD | Forward-MfeI | ccccaattgatgactgattaccggctacaaccaacg |
| MYC2 pB42AD | Reverse-MfeI | ccccaattgttaaccgatttttgaaatcaaacttg |
| MYC3 pB42AD | Forward-MfeI | ccccaattgatgaacggcacaacatcatcaatcaac |
| MYC3 pB42AD | Reverse-XhoI | cccgctcgagtcaatagttttctccgactttcgtcatc |
| MYC4 pB42AD | Forward-EcoRI | ggggaattcatgtctccgacgaatgttcaagtaaccg |
| MYC4 pB42AD | Reverse-XhoI | cccgctcgagtcatggacattctccaactttctccg |
| TT8 pB42AD | Forward-EcoRI | ggggaattcatggatgaatcaagtattattc |
| TT8 pB42AD | Reverse-XhoI | cccgctcgagctatagattagtatcatgtatta |
| EGL3 pB42AD | Forward-EcoRI | ggggaattcatggcaaccggagaaaacagaac |
| EGL3 pB42AD | Reverse-XhoI | cccgctcgagttaacatatccatgcaaccc |
| GL3 pB42AD | Forward-EcoRI | ggggaattcatggctaccggacaaaacagaacaactg |
| GL3 pB42AD | Reverse-SalI | acgcgtcgactcaacagatccatgcaaccctttgaagtgct |
| MYB75 pB42AD | Forward-EcoRI | ggggaattcatggagggttcgtccaaagggctg |
| MYB75 pB42AD | Reverse-XhoI | cccgctcgagctaatcaaatttcacagtctc |
| GL1 pB42AD | Forward-EcoRI | ggggaattcatgagaataaggagaagagatg |
| GL1 pB42AD | Reverse-XhoI | cccgctcgagctaaaggcagtactcaacatc |
| TTG1 pB42AD | Forward-EcoRI | ggggaattcatggataattcagctccagattc |
| TTG1 pB42AD | Reverse-XhoI | cccgctcgagtcaaactctaaggagctgcat |
| JAZ1 nYFP | Forward | cggacaagtttgtacaaaaaagcaggctccatgtcgagttctatggaatgttc |
| JAZ1 nYFP | Reverse | cggaccactttgtacaagaaagctgggtctatttcagctgctaaaccgag |
| JAZ10-nYFP | Reverse | cggacaagtttgtacaaaaaagcaggctccatgtcgaaagctaccatagaac |
| JAZ10-nYFP | Forward | cggaccactttgtacaagaaagctgggtc ggccgatgtcggatagtaag |
| cYFP bHLH3 | Forward | cggacaagtttgtacaaaaaagcaggctccatgggtcaaaagttttgggagaatcaag |
| cYFP bHLH3 | Reverse | cggaccactttgtacaagaaagctgggtcttactgtgatagagaggcaaggagcttg |
| cYFP bHLH13 | Forward-KpnI | cggggtaccatgaatattggtcgcctagtg |
| cYFP bHLH13 | Reverse-SalI | agcgtcgacctatctacctgatgatgttc |
| cYFP bHLH14 | Forward-KpnI | cggggtaccatgtataatctcactttctctcc |
| cYFP bHLH14 | Reverse-SalI | agcgtcgacttataggcttagggttcggacaag |
| cYFP bHLH17 | Forward-KpnI | cggggtaccatgaatatgagtgatttagg |
| cYFP bHLH17 | Reverse-SalI | agcgtcgacttatatatcaccagagacctg |
| bHLH3 nYFP | Forward-KpnI | cggggtaccatgggtcaaaagttttgggag |
| bHLH3 nYFP | Reverse-SalI | ccgctcgagctgtgatagagaggcaaggag |
| bHLH13 nYFP | Forward-KpnI | cggggtaccatgaatattggtcgcctagtg |
| bHLH13 nYFP | Reverse-XhoI | agcgtcgactctacctgatgatgttcttg |
| bHLH14 nYFP | Forward-KpnI | cggggtaccatgtataatctcactttctctcc |
| bHLH14 nYFP | Reverse-SalI | agcgtcgactaggcttagggttcggacaag |
| bHLH17 nYFP | Forward-KpnI | cggggtaccatgaatatgagtgatttagg |
| bHLH17 nYFP | Reverse-SalI | agcgtcgactatatcaccagagacctgtg |
| cYFP-TT8 | Forward | cggacaagtttgtacaaaaaagcaggctccatggatgaatcaagtattattc |
| cYFP-TT8 | Reverse | cggaccactttgtacaagaaagctgggtcctatagattagtatcatg |
| cYFP-EGL3 | Forward | cggacaagtttgtacaaaaaagcaggctccatggcaaccggagaaaacagaac |
| cYFP-EGL3 | Reverse | cggaccactttgtacaagaaagctgggtcttaacatatccatgcaaccctttg |
| cYFP-GL3 | Forward | cggacaagtttgtacaaaaaagcaggctccatggctaccggacaaaacagaac |
| cYFP-GL3 | Reverse | cggaccactttgtacaagaaagctgggtctcaacagatccatgcaaccctttg |
| cYFP-MYB75 | Forward | cggacaagtttgtacaaaaaagcaggctccatggagggttcgtccaaagggctg |
| cYFP-MYB75 | Reverse | cggaccactttgtacaagaaagctgggtcctaatcaaatttcacagtctctc |
| cYFP-GL1 | Forward | cggacaagtttgtacaaaaaagcaggctccatgagaataaggagaagagatg |
| cYFP-GL1 | Reverse | cggaccactttgtacaagaaagctgggtcctagtgatgttgagtactgccttc |
| GFP bHLH3 | Forward- SmaI | aactgcagatgggtcaaaagttttgggagaatc |
| GFP bHLH3 | Reverse-BamHI | cgcggatccttactgtgatagagaggcaaggagcttg |
| GFP bHLH13 | Forward- SmaI | aactgcagatgaatattggtcgcctagtgtggaac |
| GFP bHLH13 | Reverse- BamHI | cgcggatccctatctacctgatgatgttcttgactg |
| GFP bHLH14 | Forward-SmaI | aactgcagatgtataatctcactttctctccatc |
| GFP bHLH14 | Reverse- BamHI | cgcggatccttataggcttagggttcggacaagtgtg |
| GFP bHLH17 | Forward-EcoRI | ccggaattcatgaatatgagtgatttaggttggga |
| GFP bHLH17 | Reverse- SmaI | aactgcagttatatatcaccagagacctgtgaac |
| pbHLH3::GUS | Forward-PstI | aactgcagccagtaatgtctttctggcaataatcg |
| pbHLH3::GUS | Reverse- BamHI | cgcggatccctcctgaaacgttcattgacatggaat |
| pbHLH13::GUS | Forward-PstI | aactgcagcatacgatgagctttaagttttggtc |
| pbHLH13::GUS | Reverse- BamHI | cgcggatcccaacaaaaaggaaagacttttaag |
| pbHLH14::GUS | Forward-PstI | aactgcagcctatgtcgatatgcgccgagaaggac |
| pbHLH14::GUS | Reverse- BamHI | cgcggatccctttccagtgatcccgaccaaagaga |
| pbHLH17::GUS | Forward-PstI | aactgcaggaggtatataaatgaacggtaacact |
| pbHLH17::GUS | Reverse- BamHI | cgcggatccccagattacaccaaatcctaccaaacc |
| DB bHLH3 | Forward-SmaI | tcccccggggatgggtcaaaagttttgggag |
| DB bHLH3 | Reverse-XhoI | ccgctcgagtcaattaccattcaataaatgc |
| DB bHLH13 | Forward- SmaI | tcccccggggatgaatattggtcgcctagtgtg |
| DB bHLH13 | Reverse-SalI | acgcgtcgacctatctacctgatgatgttcttgac |
| DB bHLH14 | Forward- SmaI | tcccccggggatgtataatctcactttctctcc |
| DB bHLH14 | Reverse-SalI | acgcgtcgacttataggcttagggttcggacaag |
| DB bHLH17 | Forward- SmaI | tcccccggggatgaatatgagtgatttaggttg |
| DB bHLH17 | Reverse-SalI | acgcgtcgacttatatatcaccagagacctgtg |
| DB MYC2 | Forward- SmaI | tcccccgggggatgactgattaccggctacaaccaacg |
| DB MYC2 | Reverse- SmaI | tcccccggggttaaccgatttttgaaatcaaacttgctc |
| JAZ1-pGreenII 62-SK | Forward-SacI | atcgagctcatgtcgagttctatggaatg |
| JAZ1-pGreenII 62-SK | Reverse-SalI | agagtcgactcatatttcagctgctaaac |
| DFRpro-pGreenII 0800-LUC | Forward-KpnI | cggggtaccatttaagcttttccaagattta |
| DFRpro- pGreenII 0800-LUC | Reverse-BamHI | cgcggatccttttgtggttatatgatagattg |
| TAT1pro-pGreenII 0800-LUC | Forward-KpnI | cggggtacccattaggtatagctagactggtg |
| TAT1pro-pGreenII 0800-LUC | Reverse-BamHI | cgcggatcctctattagattttgtttttggg |
| MYB75- pGreenII 62-SK | Forward-SacI | atcgagctcatggagggttcgtccaaag |
| MYB75- pGreenII 62-SK | Reverse-SalI | agagtcgacctaatcaaatttcacagtc |
| TT8- pGreenII 62-SK | Forward-SmaI | agacccgggatggatgaatcaagtattattc |
| TT8- pGreenII 62-SK | Reverse-SalI | agagtcgacctatagattagtatcatgta |
| MYC2- pGreenII 62-SK | Forward-SmaI | agacccgggatgactgattaccggctac |
| MYC2- pGreenII 62-SK | Reverse-KpnI | cggggtaccttaaccgatttttgaaatc |
| bHLH3- pGreenII 62-SK | Forward-SmaI | agacccgggatgggtcaaaagttttgggagaatc |
| bHLH3- pGreenII 62-SK | Reverse-XhoI | ccgctcgagttactgtgatagagaggcaaggagc |
| bHLH17-pGreenII 62-SK | Forward-SmaI | agacccgggatgaatatgagtgatttag |
| bHLH17- pGreenII 62-SK | Reverse-SalI | agagtcgacttatatatcaccagagacctg |
| bHLH13 Overexpresion | Forward-SalI | acgcgtcgacatgaatattggtcgcctagtgtgg |
| bHLH13 Overexpresion | Reverse-SpeI | cggactagtctatctacctgatgatgttcttgac |
| bHLH17 Overexpresion | Forward-SalI | acgcgtcgacatgaatatgagtgatttagg |
| bHLH17 Overexpresion | Reverse-SpeI | cggactagtttatatatcaccagagacctg |
| myc-bHLH3 | Forward-SmaI | tcccccgggatgggtcaaaagttttgggag |
| myc-bHLH3 | Reverse-SmaI | tcccccgggttactgtgatagagaggc |
