# Supplementary material for: The bHLH Subgroup IIId Factors Negatively Regulate Jasmonate-Mediated Plant Defense and Development
Source: PLoS Genet. 2013 Jul 25;9(7):e1003653. doi: 10.1371/journal.pgen.1003653 (PMC3723532; doi:10.1371/journal.pgen.1003653)
Supplement: Table S2 — Primers Used for Quantitative Real-time PCR Analysis and ChIP PCR. (DOC) [file pgen.1003653.s006.doc]

**Table S2. Primers Used for Quantitative Real-time PCR Analysis and ChIP PCR.**

| DFR-Realtime PCR | Forward | tggtggtcggtccattcat |
| --- | --- | --- |
| DFR-Realtime PCR | Reverse | gagagagcgcggtgataagg |
| LDOX-Realtime PCR | Forward | tccgggtttgcagcttttc |
| LDOX-Realtime PCR | Reverse | atcaggaacacattttgcagtga |
| UF3GT-Realtime PCR | Forward | tggaggtggcggttgaa |
| UF3GT-Realtime PCR | Reverse | ctttgccgcgagaacca |
| Actin8-Realtime PCR | Forward | tcagcactttccagcagatg |
| Actin8-Realtime PCR | Reverse | ctgtggacaatgcctggac |
| VSP1-Realtime PCR | Forward | acgtccagtcttcggcatcc |
| VSP1-Realtime PCR | Reverse | tagttgatggacagtccctc |
| LOX2-Realtime PCR | Forward | cctcagatcgagtctgttc |
| LOX2-Realtime PCR | Reverse | agcagaggtaagaggccag |
| PDF1.2-Realtime PCR | Forward | tttgctgctttcgacgcac |
| PDF1.2-Realtime PCR | Reverse | gattcttgcatgcattactg |
| Thi2.1-Realtime PCR | Forward | tgctcataatgagtctggtc |
| Thi2.1-Realtime PCR | Reverse | tacaccttcccttggaaaac |
| ERF1-Realtime PCR | Forward | ttcccttcaacgagaacga |
| ERF1-Realtime PCR | Reverse | gtttgttgcgtggactgct |
| bHLH3-Realtime PCR | Forward | ggctgcaccgctgagcagttg |
| bHLH3-Realtime PCR | Reverse | gttggcttgcaatagtacattg |
| bHLH13-Realtime PCR | Forward | cgagttcaagggcttcagag |
| bHLH13-Realtime PCR | Reverse | ccactgcatctgcccatt |
| bHLH14-Realtime PCR | Forward | cctcttctttgctctcttttacaca |
| bHLH14-Realtime PCR | Reverse | aatccggaggagaagaggac |
| bHLH17-Realtime PCR | Forward | cagagaaaagaccagtgagcttg |
| bHLH17-Realtime PCR | Reverse | gtctcttctcatcaacaacagaaacta |
| bHLH3-Realtime PCR | Pair1Forward | ggaatcgtatgaggtgcaagcg |
| bHLH3-Realtime PCR | Pair1Reverse | gccgttccgcttccacatgg |
| bHLH3-Realtime PCR | Pair2Forward | ggctgcaccgctgagcagttg |
| bHLH3-Realtime PCR | Pair2Reverse | gttggcttgcaatagtacattg |
| bHLH13-Realtime PCR | Pair1Forward | cagagatcgttaggattctaag |
| bHLH13-Realtime PCR | Pair1Reverse | gccaccaaacaaatcatgaagc |
| bHLH13-Realtime PCR | Pair2Forward | cgagttcaagggcttcagag |
| bHLH13-Realtime PCR | Pair2Reverse | ccactgcatctgcccatt |
| bHLH14-Realtime PCR | Pair1Forward | cctcttctttgctctcttttacaca |
| bHLH14-Realtime PCR | Pair1Reverse | aatccggaggagaagaggac |
| bHLH14-Realtime PCR | Pair2Forward | ggcgacgaaagagaaacatc |
| bHLH14-Realtime PCR | Pair2Reverse | cgctttgtccattcgtgacac |
| bHLH17-Realtime PCR | Pair1Forward | ccgtttaagtggttttgggtt |
| bHLH17-Realtime PCR | Pair1Reverse | catcatcccaacctaaatcac |
| bHLH17-Realtime PCR | Pair2Forward | cagagaaaagaccagtgagcttg |
| bHLH17-Realtime PCR | Pair2Reverse | gtctcttctcatcaacaacagaaacta |
| DFRpro-ChIP | Forward | caacaaaatacacacctaaggaaat |
| DFRpro-ChIP | Reverse | tatatgatagattgtgctttgggaa |
| DFR_3UTR_-ChIP | Forward | gttaatcataatttatcattgg |
| DFR_3UTR_-ChIP | Reverse | ggttaattcataccgttacaatc |
| TAT1pro-ChIP | Forward | ccgaaggctcgagattcgagatac |
| TAT1pro-ChIP | Reverse | ggttttcacattgttctaaaca |
| TAT1_3UTR_-ChIP | Forward | catatatacatctcttaactac |
| TAT1_3UTR_-ChIP | Reverse | ctgggtttcttcataatttttctaa |
